# OneProt: Towards multi-modal protein foundation models via latent space alignment of sequence, structure, binding sites and text encoders

**DOI:** 10.1371/journal.pcbi.1013679

**Published:** 2025-11-13

**Authors:** Klemens Flöge, Srisruthi Udayakumar, Johanna Sommer, Marie Piraud, Stefan Kesselheim, Vincent Fortuin, Stephan Günnemann, Karel J. van der Weg, Holger Gohlke, Erinc Merdivan, Alina Bazarova

**Affiliations:** 1 PriorLabs, Berlin, Germany; 2 Helmholtz AI, Munich, Germany; 3 Independent Researcher, Coimbatore, Tamil Nadu, India; 4 School of Computation, Information and Technology, Technical University of Munich, Garching, Germany; 5 Munich Data Science Institute, Technical University of Munich, Garching, Germany; 6 Helmholtz Munich, Neuherberg, Germany; 7 Jülich Supercomputing Centre, Forschungszentrum Jülich, Jülich, Germany; 8 Munich Center for Machine Learning, Munich, Germany; 9 Institute of Bio- and Geosciences (IBG-4: Bioinformatics), Forschungszentrum Jülich, Jülich, Germany; 10 Institute for Pharmaceutical and Medicinal Chemistry, Heinrich Heine University Düsseldorf, Düsseldorf, Germany; Xinjiang Technical Institute of Physics and Chemistry, CHINA

## Abstract

Recent advances in Artificial Intelligence have enabled multi-modal systems to model and translate diverse information spaces. Extending beyond text and vision, we introduce OneProt, a multi-modal Deep Learning model for proteins that integrates structural, sequence, text, and binding site data. Using the ImageBind framework, OneProt aligns the latent spaces of protein modality encoders in a lightweight fine-tuning scheme that focuses on pairwise alignment with sequence data, rather than requiring full matches. This novel approach comprises a mix of Graph Neural Networks and transformer architectures. It demonstrates good performance in retrieval tasks and showcases the efficacy of multi-modal systems in Protein Machine Learning through a broad spectrum of downstream baselines, including enzyme function prediction and binding site analysis. Furthermore, OneProt enables the transfer of representational information from specialized encoders to the sequence encoder, enhancing capabilities for distinguishing evolutionarily related and unrelated sequences and exhibiting representational properties where evolutionarily related proteins align in similar directions within the latent space. In addition, we extensively investigate modality ablations to identify the encoders that contribute the most to predictive performance, highlighting the significance of the binding site encoder, which has not been used in similar models previously. This work expands the horizons of multi-modal protein models, paving the way for transformative applications in drug discovery, biocatalytic reaction planning, and protein engineering.

## Author summary

In this study, we introduce OneProt, a novel, versatile Artificial Intelligence system designed for protein analysis. In order to integrate different types of data, structural, sequence, text, and binding sites, OneProt uses the ImageBind framework, efficiently aligning protein data without needing full matches. Combining Graph Neural Networks and transformer architectures, OneProt excels in tasks like enzyme function prediction and binding site analysis. It enhances the understanding of protein relationships by transferring information between different data types, making it easier to identify related proteins. The OneProt framework stands out for two key features: the ability to incorporate custom modalities during pre-training and a simple fine-tuning process that requires only a Multi-Layer Perceptron projection. Notably, we also show that incorporating multiple modalities can reduce the need for extensive datasets and training, leading to competitive downstream performance. In addition, we conduct an exhaustive ablation study, where we highlight the crucial role of the binding site encoder, which has not been used in similar models before. Overall, OneProt represents a significant step forward in multi-modal protein modeling, with promising applications in drug discovery and protein engineering.

## Introduction

The protein space is vast and high-dimensional; even for a 100-residue polypeptide chain, there are $∼10^{130}$ possible sequences [1]. The topological space of functional and synthesizable proteins is significantly smaller, but identifying these meaningful subspaces using experimental methods remains challenging. While machine learning methods for investigating proteins are not new [2], recent increases in computing power [3], advances in algorithms, and the availability of extensive sequence data [4] have collectively sparked a revolution in computational protein design [5–7]. However, challenges such as achieving tunable control over protein conformations and ensuring precise shape complementarity for molecular recognition are becoming feasible only now [8].

In recent years, multi-modal Artificial Intelligence (AI) systems and foundation models have notably become more prominent. Initially introduced for text-to-image tasks, the CLIP framework [9], which efficiently learns visual concepts from natural language supervision, has been adapted to various architectures. This evolution is evident in the transition from the multi-modal capabilities of GPT-3 [10] to GPT-4 [11]. Furthermore, ImageBind [12] demonstrated that aligning pairs of modalities is sufficient to unify the latent space of all modalities, provided one of the paired modalities is consistently present.

While powerful, sequence-only protein language models can struggle to capture functional properties dictated by three-dimensional structure or explicit evolutionary constraints. As highlighted in [13], extending models to include additional information beyond sequences is a promising direction for learning richer protein representations. Integrating complementary modalities addresses these gaps: tools for sequence alignment [14] provide evolutionary context, and binding site prediction [15] directly identifies key interaction interfaces. The enriched multi-modal representations support a range of downstream tasks, including protein function prediction [16] and enzyme or antibody design [13], thereby advancing functional protein design [17].

Building on the success of evolutionary scale modeling (ESM) [18–20], recent studies show that integrating these models with modality-specific encoders enhances performance in protein-related tasks. This multi-modal synergy is powerful because it allows a model to, for instance, jointly reason about a residue’s sequence context, its structural environment, and its evolutionary conservation. This leads to better performance in tasks such as enzyme function prediction and antibody stability [21,22], showcasing the potential of multi-modal approaches for accurate protein function and interaction predictions. In parallel, large-scale sequence-based architectures such as AIDO [23] demonstrate the scalability of mixture-of-experts designs, which, while unimodal, reflect the same principle of distributed specialization that underlies multi-modal fusion. However, it is worth noting that such expansive models trained on vast amounts of data have faced criticism, highlighting that an efficient codebase and careful curation of training data can yield better results even with smaller models [24]. Moreover, large models are expensive to train and often require significant optimization to perform inference.

Multi-modal AI approaches have been explored in several innovative ways in the molecular sciences, including 3D molecule-text modalities in language models [25], the combination of molecule graphs with natural language [26], and text-protein sequence alignment [27]. Structural information has been successfully incorporated into sequence vocabularies [28]. In addition, retrieval systems have been developed to query molecular structures based on text descriptions [29] and to generate target molecules based on protein pockets [30]. The models developed by [31], [32] are built using the ImageBind framework. The latter, ProTrek, focuses on all possible pairs of modalities during training and demonstrates retrieval results superior to classical bioinformatics search engines, delivering strong performance in several downstream tasks, using exclusively transformer-based architectures. On the other hand, [33] employs a knowledge graph-based approach for modality alignment.

**Fig 1:**
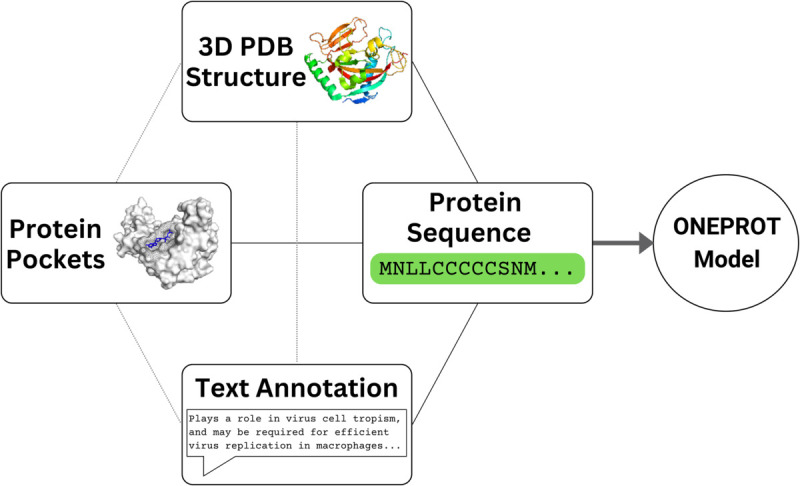
Overview of OneProt’s alignment of protein sequences with other modalities for comprehensive cross-modal integration. Training is performed using pairs comprising a sequence and another modality, leading to the emergent alignment between all other modalities, as indicated by the dashed lines.

In this manuscript, we introduce OneProt, Fig 1, which effectively extends the ImageBind framework to the protein space using transformer-based and Graph Neural Network (GNN) encoders. Our model aligns protein sequences, protein structures represented in two ways, protein pockets, and text annotations by pairing each of the latter modalities with the sequence modality only. We curate the respective training datasets using publicly available databases. Moreover, to reduce redundancy, we focus on aligning the core modalities by clustering the sequences at ≤50% identity. OneProt’s multi-modal alignment enables efficient, unbiased retrieval and downstream tasks using a comprehensive protein dataset. Its flexible and versatile framework also allows one to easily add new modalities to the model, if necessary. We conduct extensive ablations to evaluate each modality’s contribution to downstream tasks, addressing a gap left by similar models. Based on performance, we introduce two models: OneProt-5 (five modalities) and OneProt-4 (four modalities). Importantly, their moderate size allows for a seamless application to new downstream tasks when needed.

## Materials and methods

### Representation alignment

**Fig 2:**
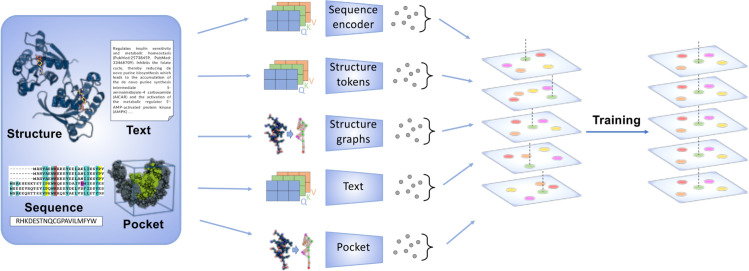
Overview of the OneProt model. The model aligns multiple modalities, including primary protein sequence, 3D protein structure, binding pockets, and text annotations. Each modality is processed by its respective encoder, generating embeddings aligned in a shared latent space, facilitating cross-modal learning and integration.

Illustrated in Figs 1 and 2, the training data of the OneProt model consists of paired samples from multiple modalities, each contributing unique information for a comprehensive protein representation. In modality pairs (ℱ,ℰ), ℱ always denotes the protein sequence, which is consistently present in each tuple, while ℰ can represent the structure, text, or pocket modality. A sample pair $\left(a_{i},b_{i}\right)$ consists of data points from two different modalities of the same protein, where i∈{1…n} are the indices within a batch of size *n*. Representations ${a^{\prime }}_{i}$ and ${b^{\prime }}_{i}$ are obtained using their respective encoders $\phi_{ℱ}$ and $\phi_{ℰ}$: ${a^{\prime }}_{i}=\phi_{ℱ}(a_{i})$ and ${b^{\prime }}_{i}=\phi_{ℰ}(b_{i})$, where $\phi_{ℱ}:ℱ\to {\mathbb{R}}^{m}$, and $\phi_{ℰ}:ℰ\to {\mathbb{R}}^{k}$, and therefore ${a^{\prime }}_{i}$ and ${b^{\prime }}_{i}$ are numeric vectors of the dimensions *m* and *k*, *m* not necessarily equal *k*. Then, in order to align the latent spaces of the encoders, projection heads $proj_{ℱ}:{\mathbb{R}}^{m}\to {\mathbb{R}}^{l}$ and $proj_{ℰ}:{\mathbb{R}}^{k}\to {\mathbb{R}}^{l}$ are applied to ${a^{\prime }}_{i}$ and ${b^{\prime }}_{i}$ respectively, thereby mapping them int the shared space ${\mathbb{R}}^{l}$. The projected vectors $proj_{ℱ}(a_{i}^{\prime })$ and $proj_{ℰ}(b_{i}^{\prime })$ are then *L*_2_-normalized to produce final unit embeddings ${𝐚}_{i}=proj_{ℱ}(a_{i}^{\prime })/||proj_{ℱ}(a_{i}^{\prime })|{|}_{2}$ and ${𝐛}_{i}=proj_{ℰ}(b_{i}^{\prime })/||proj_{ℰ}(b_{i}^{\prime })|{|}_{2}$.

Given a batch of pairs $\left\{(a_{1},b_{1}),...,(a_{n},b_{n})\right\}$, the goal is to synchronize the representations of ${𝐚}_{i}$ and ${𝐛}_{i}$ (positive pairs) while pushing ${𝐚}_{i}$ and ${𝐛}_{j}$ apart for i≠j (negative pairs). Following [9], the InfoNCE loss [34] is defined as:

$$L_{ℱ,ℰ}=-\frac{1}{n}\sum_{i}\log\frac{\exp\left({𝐚}_{i}^{⊤}{𝐛}_{i}/\tau \right)}{\exp\left({𝐚}_{i}^{⊤}{𝐛}_{i}/\tau \right)+\sum_{j\neq i}\exp\left({𝐚}_{i}^{⊤}{𝐛}_{j}/\tau \right)}$$ (1)

where ${𝐚}_{i}^{⊤}{𝐛}_{i}$ is a dot-product in ${\mathbb{R}}^{l}$ between the normalized vectors ${𝐚}_{i}$ and ${𝐛}_{i}$, reflecting therefore cosine similarity between them, and *τ* is the temperature parameter. Small values of *τ* lead to larger-magnitude arguments in the exponent (logits). As a result, even small differences in similarity between the positive pair and the negatives translate into large differences in probability, enforcing stronger separation. Conversely, large values of *τ* shrink the logits, flattening the softmax distribution. This reduces sensitivity to differences in similarity, making the separation between positive and negative pairs less pronounced. The total loss, based on the loss in (1), is computed while considering the order of the modalities as follows:

$$L_{\text{total}}=L_{ℱ,ℰ}\;+\;L_{ℰ,ℱ}.$$ (2)

This symmetric formulation ensures bidirectional alignment, so the model learns both to retrieve ℰ given ℱ and vice versa, following the CLIP-style contrastive setup.

In line with the ImageBind framework, we construct batches of paired modalities $\left(ℱ,{ℰ}_{1}),\ldots ,(ℱ,{ℰ}_{n}\right)$, consistently including the sequence modality ℱ in each batch as an anchor. For each pair $\left(ℱ,{ℰ}_{i}\right)$ we compute the contrastive loss and immediately apply a gradient update of the respective encoders and projection heads. This sequential optimization differs from the original CLIP training, which aggregates losses across a fixed two-modality pair (image–text) and updates once per batch. This approach ensures the alignment of the sequence modality with every other modality. Moreover, because all modalities are mapped into the same shared latent space via ℱ, modalities, which were not directly paired together during training, still align with each other - this is also known as emergent alignment, as illustrated in Fig 2.

### Model details

OneProt integrates pre-trained models across various modalities when available. Otherwise, the corresponding encoder is trained from scratch using the loss as in Eqs (1), (2). In what follows, we elaborate on the choice of the respective encoders, their suitability for our tasks, and architectural details. The training methodology is summarized in Table 1: we employ a mix of frozen, fully trained, and Low-Rank Adaptation (LoRA) [35] methods to optimize performance across modalities.

**Table 1:** Overview of OneProt’s different encoders.

Modality	Model	Training	Pooler	Projection	Full Parameter Count	Trainable Parameter Count
Sequence	ESM2	Freeze	attention	linear	652 M	0
Structure-PDB	ProNet	Full	-	linear	2.6 M	2.6 M
Structure-Token	ESM2	Full	mean	linear	35 M	35 M
Pocket	ProNet	Full	-	linear	2.6 M	2.6 M
Text	MSR BiomedBERT	LoRA	cls	MLP	110 M	0.4 M
Total:					802.2 M	40.6 M

The models described in this manuscript and the Supporting information were pre-trained using 64 NVIDIA A100 GPUs (16 compute nodes) of the JUWELS Booster supercomputer [36] for 33,000 optimizer steps using Distributed Data Parallel (DDP) scheme. Further information on the pre-training is available in S1 Text.

#### Sequence and structure token encoder.

ESM2 is a transformer-based architecture, which serves as a standard foundation model for the sequence modality, having demonstrated superior performance across a wide range of protein-related tasks [18,37,38]. Therefore, we use ESM2 650M [37] for the common modality sequence with its pre-trained representations as a stable reference point to anchor the other modalities.

The respective encoder works as follows. Given a protein sequence $x\in {\mathbb{R}}^{n\times d}$, where *n* is the sequence length and *d* is the embedding dimension, the transformer-based encoder processes the input sequence through layers of self-attention and feed-forward neural networks.

The embedding for the input sequence is initialized as:


$$x_{0}=E(x)\;+\;P(x)$$

where *E*(*x*) denotes the learned embedding for each amino acid in the sequence, and *P*(*x*) represents the positional encoding to capture the sequential nature of the data. This embedding is then processed through multiple layers of the transformer model.

Each transformer layer consists of two main components: multi-head self-attention and a position-wise feed-forward network. For a single transformer layer, the update is given as:


$$\text{MultiHead}(\{Q_{i},K_{i},V_{i}{\}}_{i=1}^{p})=\text{Concat}({\text{head}}_{1},{\text{head}}_{2},\ldots ,{\text{head}}_{p})W^{O}$$

where $Q_{i}=x_{0}W_{i}^{Q}$, $K_{i}=x_{0}W_{i}^{K}$, and $V_{i}=x_{0}W_{i}^{V}$ represent the query, key, and value matrices, and each head performs scaled dot-product attention:


$${\text{head}}_{i}=\text{softmax}\left(\frac{Q_{i}K_{i}^{T}}{\sqrt{d_{k}^{i}}}\right)V_{i}$$

where all *W* are learnable parameter matrices, and $W_{i}^{K}\in {\mathbb{R}}^{d\times d_{k}^{i}}$. The attention mechanism captures dependencies across the sequence by allowing each position to attend to all others. Following the attention block, the position-wise feed-forward network updates the representation as:


$$\text{FFN}(h)=\max(0,hW_{1}\;+\;b_{1})W_{2}\;+\;b_{2}$$

where *W*_1_ and *W*_2_ are learned weight matrices and *h* is the output of the multi-head self-attention.

The transformer updates the sequence representation through stacked layers of multi-head self-attention and position-wise feed-forward networks:


$$h_{\text{seq}}^{\left(l+1\right)}=\text{TransformerLayer}(h_{\text{seq}}^{\left(l\right)}),\;l=1,2,\ldots ,L$$

where *L* is the number of layers, and $h_{\text{seq}}^{\left(l\right)}\in {\mathbb{R}}^{n\times d}$ represents the sequence embeddings at the *l*-th layer.

The final output of the sequence transformer is:


$$h_{\text{seq}}=h_{\text{seq}}^{\left(L\right)}$$

which provides the learned sequence representations for downstream tasks, capturing local and global dependencies across the protein sequence.

To capture the broader structural organization of a protein from a discrete 3D token representation, we used structure tokens as proposed in [28] to encode the structure modality, and trained the aforementioned transformer architecture from scratch, given the lack of a suitable pre-trained model for the structure modality.

The Multiple Sequence Alignment (MSA) modality has also been efficiently implemented via the transformer-based ESM-1b model [19], however, this modality was not included in the final training due to resulting in a significant speed decline and high memory consumption.

#### Graph structure and pocket encoder.

To allow the network to capture detailed chemical information of the protein structure, we train the all-atom ProNet graph model [39], a strong performer in graph-based protein modeling, from scratch, due to the absence of a suitable pre-trained model.

The ProNet encoder operates by modeling protein structures as hierarchical 3D graphs to capture relationships at multiple levels of granularity: amino acid, backbone, and all-atom levels. Each amino acid is represented as a node, and edges between nodes are defined by a cut-off radius. This hierarchical representation is particularly valuable for multi-modal learning, as it enables the model to align structural features at different scales with complementary modalities—for instance, potentially matching local atomic interactions with sequence motifs.

A protein graph G=(V,E,P) is constructed, where *V* represents nodes for aminoacids, *E* denotes edges for interactions between them, and *P* contains the positional information of the atoms in each amino acid. ProNet incorporates a complete geometric representation *F*(*G*), for each level, to effectively learn the hierarchical structures of proteins. At the amino acid level, ProNet constructs a coarse-grained representation using the coordinates of $C_{\alpha }$ atoms, capturing the amino acid’s overall position and orientation relative to its neighbors.

To represent the all-atom structure, ProNet computes Euler angles ($\tau_{1},\tau_{2},\tau_{3}$) between the planes of neighboring amino acids. These angles capture the rotational degrees of freedom between adjacent amino acids. At the all-atom level, side-chain torsion angles ($\chi_{1},\chi_{2},\chi_{3},\chi_{4}$) provide fine-grained details of each amino acid’s side chain.

The hierarchical message passing in ProNet is governed by the equation:


$$v_{i}^{l+1}=\text{UPDATE}\left(v_{i}^{l},\sum_{j\in N_{i}}\text{MESSAGE}(v_{j}^{l},e_{ij},F(G))\right)$$

where $v_{i}^{l}$ is the feature vector of node *i* at layer *l*, *N*_*i*_ denotes the neighbors of node *i*, and the UPDATE and MESSAGE functions process the node and edge features as well as geometric information from *F*(*G*).

By incorporating the complete representations at multiple levels, ProNet captures global and local structural details of proteins, enabling it to outperform other methods in various protein-related tasks. We use the ProNet implementation as presented in the DIG library [40]. To learn the local chemical environment within a protein pocket, we trained an all-atom ProNet model also for the pocket modality from scratch.

#### Text encoder.

For the text modality, we selected the transformer-based MSR BiomedBERT [41]. This model was specifically pre-trained on a large corpus of biomedical literature, making it well-suited to our dataset. It has demonstrated state-of-the-art performance on relevant tasks such as biomedical text classification [42]. We employ it to encode protein descriptions for the critical task of mapping them to the standardized controlled vocabulary from UniProt [4].

The encoder leverages the Masked Language Model (MLM) approach, where input text sequences $x=(x_{1},x_{2},\ldots ,x_{n})$ are partially masked, and the model predicts the masked tokens *x*_*m*_. The training objective is to minimize the cross-entropy loss between the predicted tokens ${\hat{x}}_{m}$ and the original masked tokens *x*_*m*_:


$${ℒ}_{\text{MLM}}=-\sum_{m\in ℳ}\log P(x_{m}|x_{⧵m};\theta ),$$

where ℳ denotes the set of masked positions, $x_{⧵m}$ represents the input sequence with the *m*-th token masked out, and *θ* are the model parameters. This technique enables the model to learn deep contextualized representations, ${𝐡}_{i}$, for each token *x*_*i*_. By incorporating domain-specific corpora, such as PubMed abstracts, and using specialized vocabulary derived from in-domain text, the model is fine-tuned to understand complex biomedical language effectively.

We adapt the text modality to align more closely with the sequence embeddings by applying LoRA, which provides efficient fine-tuning while preserving most of the pre-trained weights. Namely, the weight matrix $W^{\prime }$ of the network is represented as $W^{\prime }=W\;+\;\frac{\alpha }{r}\Delta W=W\;+\;\frac{\alpha }{r}AB$, where $W\in {\mathbb{R}}^{n\times m}$ is the frozen original weight matrix of the pre-trained model, $A\in {\mathbb{R}}^{n\times r}$, $B\in {\mathbb{R}}^{r\times m}$ are the matrices of low rank r≪min(n,m), which are updated during the gradient descent algorithm, and *α* is a scaling parameter, controlling the contribution of the low-rank updates.

#### Projection head.

To enable contrastive learning on the embedding spaces of the same dimension, we implement a fully trainable projection layer on top of each protein encoder’s latent space. This ensures that the latent spaces of different encoders are aligned and compatible for downstream tasks. The encoders vary in pooling and projection methods to suit the specifics of each modality, as summarized in Table 1.

Specifically, for sequence modality, we adopt the Attention1D Pooling Head mechanism, inspired by [43]. The projection head employs a 1D convolution operation, which can be described as follows: given an input sequence $x\in {\mathbb{R}}^{N\times L\times C_{\text{in}}}$, where *N* is the batch size, *L* is the sequence length, and $C_{\text{in}}$ is the number of input channels, we apply a convolutional filter $w\in {\mathbb{R}}^{C_{\text{out}}\times C_{\text{in}}\times K}$, where $C_{\text{out}}$ is the number of output channels and *K* is the kernel size.

The convolution operation for each position *t* in the sequence and output channel *j* can be expressed as:

$$y_{t,j}=\sum_{i=1}^{C_{\text{in}}}\sum_{k=0}^{K-1}w_{j,i,k}\cdot x_{t+k,i}$$ (3)

where *w*_*j*,*i*,*k*_ represents the filter weights and *x*_*t* + *k*,*i*_ is the input at the corresponding position and channel.

To handle variable-length sequences and focus on relevant parts of the input, we incorporate a masked convolution approach. This involves applying a binary mask $m\in {\mathbb{R}}^{N\times L\times 1}$ to the input:

$$y_{t,j}=\sum_{i=1}^{C_{\text{in}}}\sum_{k=0}^{K-1}w_{j,i,k}\cdot (x_{t+k,i}\cdot m_{t+k})$$ (4)

This masking technique ensures that the convolution operation only considers valid positions in the sequence, effectively handling padding and improving the model’s ability to focus on meaningful information.

The Attention1D Pooling Head further refines the output by applying an attention mechanism, allowing the model to dynamically weight different parts of the sequence based on their importance for the downstream task.

### Data preparation

Our dataset combines the OpenFold training database [44] with UniProtKB/Swiss-Prot [45]. Using MMseqs2 [14], we cluster sequences at 50% identity, to balance, on the one hand, that each cluster represents a homologous group in the protein fold space and, on the other hand, to ensure that clustered proteins share evolutionary relationships. We align the training, validation, and test splits by these sequence clusters. Leveraging the unique UniProtAC identifier of each protein sequence, we filter the MSAs from OpenFold according to the UniProtACs, and locate a structure in the AlphaFold2DB for each one [46]. To obtain the structure tokens, we use the SaProt [28] training dataset, removing sequences with less than 50% sequence identity to the validation and test sets. We then randomly sub-sample a million entries and mask the sequence tokens to extract structure tokens for each UniProtAC. Using P2Rank [15], we predict the binding site for each structure, where possible, resulting in fewer entries than for the structure modality. We create text annotations by mining the UniProt dataset for the keywords associated with the corresponding UniProtACs. Dataset sizes are listed in Table 2. Full details on dataset creation can be found in the Supporting information, S3 Text.

**Table 2:** OneProt data overview.

Modality	Dataset Size
Sequence	1.04 M
Structure PDB	656 K
Structure Tokens	1 M
Pocket	341 K
Text	546 K

### Downstream evaluation

#### Modality alignment.

After aligning modalities (section Representation alignment) and training OneProt using the symmetrical loss function (Eq (2)), we evaluate the alignment of latent spaces by constructing a vector database from paired test datasets and conducting cross-modality similarity searches, thereby verifying that representations of the same protein are consistently proximate across different modalities.

To assess modality alignment, we evaluate each modality’s retrieval performance against the sequence reference modality. For *n* modalities, we define 2×(n−1) cross-modal retrieval tasks from the sequence modality, encompassing both forward and reverse directions. Additionally, we introduce emergent retrieval tasks to evaluate modality pairs not directly trained together, resulting in (*n*–1)(*n*–2) tasks for *n* modalities.

For each modality pair, we compute R@1, R@10, and R@100, where these metrics correspond to the accuracy of the correct retrievals from the nearest, closest ten, or closest hundred embeddings in the latent space, respectively, with the accuracy value of 1 corresponding to the perfect alignment between modalities. We also compute median rank (MR), which measures how well matching representations align by ranking the cosine similarities between pairs modalities for each protein, taking the median of these ranks and averaging across all proteins. Lower values indicate better performance, with 1 representing the best possible score. More details on how MR was computed is available in S1 Text.

We use 4000 held-out modality pairs for each of the cross-modal retrieval tasks as the test set.

#### Supervised fine-tuning for downstream tasks.

We demonstrate the utility of OneProt by evaluating its performance on a range of protein-related downstream tasks, following the ones presented in [28] and using the same metrics, where the accuracy thresholds for classification tasks are fixed and chosen in a standard way. These tasks use well-established benchmarks to encompass protein structure, property, and function predictions. Details on these evaluations can be found in the S2 Text.

For the thermostability task, we use the “Human-cell” splits from FLIP [47], which were designed to predict protein thermostability values directly from sequence. This is a regression task, where the evaluation metric is a Spearman’s rank correlation coefficient *ρ* defined for paired observations $(X,Y)=\{(X_{i},Y_{i}){\}}_{i=1}^{n}$, and their respective ranks $(R(X),R(Y))=\{(R(X_{i}),R(Y_{i})){\}}_{i=1}^{n}$, with sample size *n* as follows

$$\rho =\frac{\text{cov}(R(X),R(Y))}{\sigma_{R(X)}\sigma_{R(Y)}}$$ (5)

The numerator of Eq (5) corresponds to the covariance between the ranks of (*X*,*Y*), and the denominator corresponds to the product of the respective standard deviations. The data for this task is drawn from the experimental results reported in the Meltome Atlas [48].

Second, we assess OneProt on the Human Protein-Protein Interaction (HumanPPI) task [49]. This is a binary classification task for protein pairs: positive pairs are defined as experimentally validated interactions from the Human Protein Reference Database [50], while negative pairs are constructed from proteins localized to different subcellular compartments.

Third, we evaluate the model’s performance on the Metal Ion Binding task [51], which examines a protein’s ability to bind metal ions. This, again, is a binary classification task, where proteins annotated with metal-ion binding sites in the Protein Data Bank [52] are treated as experimentally confirmed positive instances, and those lacking such annotations serve as negative instances.

Fourth, we evaluate the prediction of Enzyme Commission (EC) numbers [6]. The hierarchical EC classification system enables the prediction of enzyme functions, essential for understanding the catalytic roles of enzymes in biochemical reactions. Fifth, for a key task of functional genomics, Gene Ontology (GO) annotation [6], we evaluate OneProt’s ability to predict three types of protein function: molecular function (MF), biological process (BP), and cellular component (CC). Each of these four tasks is a multilabel classification problem with 585, 489, 1943, and 320 labels, respectively. For the evaluation, we use the $F_{\text{max}}$ score, defined as follows:


$$Precision(\tau )=\frac{1}{N}\sum_{i}^{N}prec_{i}(\tau )\;Recall(\tau )=\frac{1}{N}\sum_{i}^{N}rec_{i}(\tau )$$

$$prec_{i}(\tau )=\frac{TP_{i}(\tau )}{TP_{i}(\tau )+FP_{i}(\tau )}\;rec_{i}(\tau )=\frac{TP_{i}(\tau )}{TP_{i}(\tau )+FN_{i}(\tau )}$$

$$F_{1}(\tau )=\frac{2Precision(\tau )Recall(\tau )}{Precision(\tau )+Recall(\tau )}$$

$$F_{max}=\max_{\tau }F_{1}(\tau )$$ (6)

In Eq (6), i∈{1…N} enumerates proteins in the sample; $TP_{i},FP_{i}$, and *FN*_*i*_ correspond to the number of true positive, true negative, and false negative labels for protein *i*, respectively; *τ* is a decision threshold, such that the labels with predicted scores above *τ* are classified as positive.

Finally, the DeepLoc benchmark [53] was employed to predict the subcellular localization of a protein. It comprises two tasks: a binary classification task to determine whether a protein is membrane-bound or soluble, and a ten-class classification task that assigns each protein to a specific subcellular location. All proteins used in this downstream evaluation are experimentally annotated in the UniProt database.

For both binary and multiclass classification tasks, we report accuracy as the primary evaluation metric, following the original study [28]. For binary tasks, we additionally report the Area Under the Receiver Operating Characteristic Curve (AUC) to provide complementary evaluation.

As one of the baselines, we include the results of SaProt-LoRa from the original study [28], under the assumption that they were already optimal; consequently, mean and standard deviation are not reported for this model. This robust baseline fine-tunes a structure-aware sequence model using a Multi-Layer Perceptron (MLP) projection head for supervised learning in each downstream task, thereby updating 7-12 million parameters during the fine-tuning phase, out of which 5.4 million parameters remain in the SaProt backbone. In contrast, our approach is simpler: we keep our baseline ESM/OpenFold/SaProt/ProTrek models and OneProt frozen, generate sequence embeddings, and apply an MLP for the supervised learning problem using these embeddings. This procedure involves fine-tuning up to 1.5 million parameters, depending on the hyperparameter configuration and the output dimension, which results in faster convergence and lower memory requirements. While our setup is versatile and would support any multi-class classifier, we restrict our analysis to MLPs. This approach only requires downloading the pre-trained OneProt model to generate embeddings, enabling easier application across alternative datasets with reduced computational overhead.

Apart from ESM-2, which is a baseline encoder for our model, we have investigated the performance of a more contemporary and larger model, ESM-3, as well as the ESM-IF [54] encoder, which takes protein structures as inputs and returns the respective embeddings. Another baseline is the OpenFold model in the SoloSeq mode [55], which substitutes traditional MSA input by ESM-1b embeddings [18]. We include OpenFold as a baseline because its structure-focused architecture, offering a complementary comparison to sequence-based encoders. Note, that we provide two tri-modal ProTrek baselines which use the corresponding ESM-2 encoders with 35M and 650M parameters, overall amounting to 200 and 930 million trainable parameters, respectively, against 40.6 million trainable parameters in the OneProt backbone. Both of these utilize the contrastive learning framework but were trained on a dataset of 40M datapoints, whereas OneProt’s largest modality amounts to 1.04M datapoints. While ProTrek-35M was trained from encoder checkpoints for a duration comparable to OneProt, ProTrek-650M required nearly twice as much training time, with both ProTrek models using a more complex parallelization scheme. We do not compare OneProt’s training time with models other than ProTrek, as these were either trained from scratch, typically requiring orders of magnitude more computational resources, or, as in the case of OpenFold SoloSeq, lack publicly available details on how the ESM-1b checkpoint was adapted.

We evaluate model performance on all downstream tasks over six independent runs with random initializations and report the values corresponding to means and standard deviations across these runs. To statistically compare model performances, we conduct two types of two-sample Wilcoxon rank-sum tests, as the data did not satisfy the normality assumption. Firstly, to assess whether OneProt models outperform the baselines, we apply a one-sided test with the alternative hypothesis that OneProt performs better than the baseline. In this setting, rejecting the null hypothesis at *p* < 0.05 indicates that OneProt significantly outperforms the baseline. Secondly, to evaluate whether OneProt’s performance is comparable to the baselines, we use a two-sided test with the alternative hypothesis that the performance distributions differ. Here, failure to reject the null hypothesis implies that the performance difference between OneProt and the baseline is not statistically significant at the 0.05 level. Hereafter, we report the *p*-values from the one-sided Wilcoxon test when stating that one model outperforms another, and from the two-sided Wilcoxon test when stating that the models have comparable performance.

#### Enzyme function prediction.

To further substantiate our claim that OneProt not only aligns the latent spaces of encoders across various protein data types but also holds potential as a building block for a foundational protein model, we evaluate it on a large downstream task specifically designed to aid enzyme function prediction using the large-scale TopEnzyme database [56]. TopEnzyme is a collection of experimentally resolved (around 20,000 structures from Binding MOAD database [57]) and computationally predicted protein structures ordered by EC numbers, consisting of around 231K enzymes, with a 30% sequence identity split. EC numbers comprise a four-number hierarchy, where the first three levels represent the main-, sub-, and subsub-class functions, while the fourth level is the specific enzyme function designation.

Similarly to section Supervised fine-tuning for downstream tasks, we train a simple MLP based on the embeddings produced by the baseline and OneProt models to learn and predict EC numbers at the full hierarchy, i.e., to predict the specific enzyme function designation, overall comprising 826 classes. We compare the performance of OneProt models against several strong baselines on the EC task: ESM-2, ESM-IF, OpenFold, and ProTrek-650M, identified as top-performing models in section Supervised fine-tuning for downstream tasks. We also include ProTrek-35M in our benchmark, as it shares a similar architecture with OneProt and was trained for a comparable duration. As in previous sections, models were evaluated over six independent runs with random initializations, and the predictions from these runs were concatenated to account for variability. We also consider two deep learning models specifically designed to classify EC numbers, TopEC, [16], a graph neural network encoding a local descriptor of protein structure, and CLEAN [21], a contrastive learning model for sequence data. For these, we did not perform six independent runs; instead, we report the results provided in the original papers, where the models had already been optimized.

To assess each model’s performance across classes, we compute the Area Under Precision-Recall curve (AUPR) for each class, calculating precision and recall within the instances of that class, and then visualize the distribution over 6 runs via the corresponding boxplots.

As in the previous section, we use a one-sided two-sample Wilcoxon rank-sum test to evaluate whether OneProt outperforms the baselines (alternative hypothesis) with respect to the AUPR value distributions.

#### Representation learning for evolutionary related proteins.

Because MSA enables the detection of sequence similarity that correlates with evolutionary relatedness, we leverage the OpenProteinSet [44], which provides extensive MSA data obtained from an all-against-all search on Uniclust30 [58], to evaluate whether OneProt can provide distinct representations of evolutionary relationships compared to the baseline ESM2 and ProTrek models.

For this, following [59], we process MSA files by computing pairwise Hamming distances to rank sequences based on their similarity or dissimilarity relative to a reference sequence. Analogously to the approach used in [60], to verify the protein representation, for each reference, we select 50 most similar sequences and 50 most divergent ones, alongside 1,000 unrelated sequences as a control group. Using the ESM-2, ProTrek-35M and -650M, and two OneProt models, we generate embeddings and compute the cosine similarity between each reference sequence and its aligned (similar/divergent) or unrelated sequences. In this representation learning experiment across different models, we refrain from using SaProt, ESM-IF and OpenFold to avoid the inclusion of structural information in the input or architecture, and, therefore, only focus on the protein sequence.

This task illustrates zero-shot learning, where the models are applied to the dataset without any prior fine-tuning. To compare cosine similarity distributions, we use a one-sided Wilcoxon rank-sum test: for paired samples when comparing embeddings from different models within the same sequence class, and for unpaired samples when comparing embeddings across different sequence classes.

#### ProSPECCTs.

The ProSPECCTs (Protein Site Pairs for the Evaluation of Cavity Comparison Tools) benchmarking initiative [61] represents a significant advancement in the field of computational biology, specifically in the analysis and comparison of protein-ligand binding sites. This study meticulously developed a series of tailored benchmark datasets designed to evaluate the performance of various binding site comparison methodologies. The ProSPECCTs datasets encompass a diverse range of protein cavity pairs, enabling systematic evaluation of the strengths and limitations of different comparison tools across multiple application domains. This comprehensive framework is crucial for guiding researchers in selecting appropriate tools for specific challenges, such as drug re-purposing, promiscuity prediction, or protein-protein interactions analysis. The dataset details are provided in the S2 Table. We note, that these datasets comprise both computationally derived and experimentally determined proteins. The latter include crystallographic data (DS1, DS5 in S2 Table), Nuclear Magnetic Resonance spectroscopy data (DS2), and others.

Similar to section Representation learning for evolutionary related proteins, we demonstrate the zero-shot capabilities of OneProt here. We evaluate OneProt alongside the top-performing ProTrek models from section Supervised fine-tuning for downstream tasks, as well as the ESM-2 model, which serves as the baseline for both ProTrek and OneProt, to highlight the incremental value of incorporating additional modalities. After generating ProSPECCTs embeddings from the corresponding models, we compute cosine similarities to group the distinct pairs as defined in the ProSPECCTs dataset. We then apply threshold values to these similarity scores to generate Receiver Operating Characteristic curves, which enable classification of pairs as similar or dissimilar. Finally, we calculate Area Under Receiver Operating Curve (AUC) values.

#### Ablations.

For all downstream tasks described in the previous sections, we conduct exhaustive ablations, resulting in the evaluation of 15 different models, ESM-2 corresponding to the 16th, sequence-only ablation. We followed the same protocol of 6 independent runs, as in the sections Supervised fine-tuning for downstream tasks and Enzyme function prediction. Moreover, we visualize the performance differences between models using heatmaps for normalized performance drop $\Delta_{x,y}$ between a pair of models *x* and *y*, which is defined as

$$\Delta_{x,y}=\frac{Perf_{y}-Perf_{x}}{Perf_{x}},$$ (7)

where *Perf* corresponds to the corresponding original averaged metric (accuracy in case of binary classification) from the section Supervised fine-tuning for downstream tasks. We perform statistical testing as described in section Supervised fine-tuning for downstream tasks.

## Results

Here, we discuss the results obtained by performing downstream tasks for two OneProt models, OneProt-5 and OneProt-4, with the structure token encoder omitted in the latter. Moreover, we present the ablations and draw conclusions on the links between the modality alignment and downstream performance.

### Modality alignment

**Fig 3:**
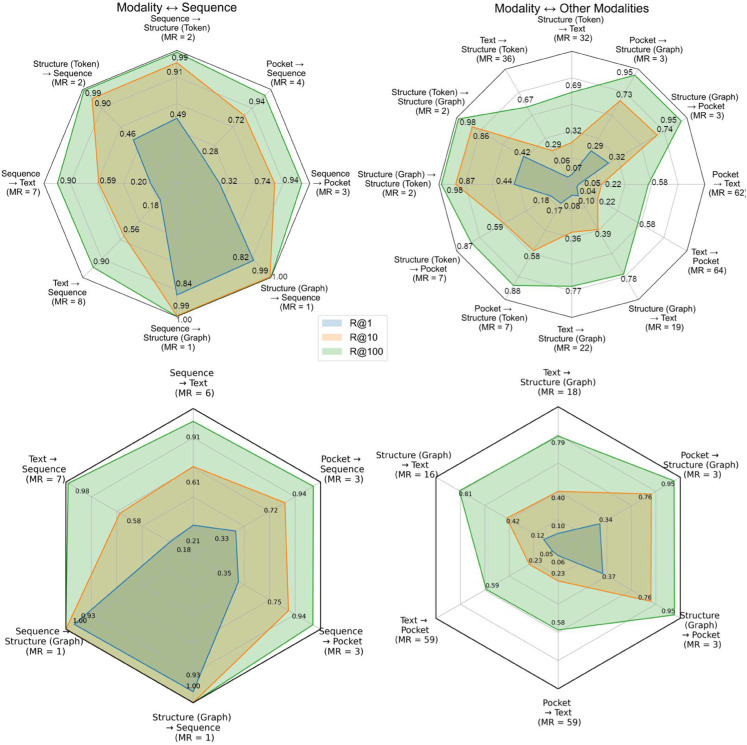
Alignment performance across modality combinations paired (left column) and not paired (emergent, right column) during training for OneProt-5 (top row) and OneProt-4 (bottom row). The axes of the polygons correspond to the modality pairs, and the vertices correspond to R@1 (inner polygon), R@10 (middle polygon), and R@100 (outer polygon), which represent the fraction of queries for which the correct (ground-truth) match appears among the top 1, top 10, or top 100 retrieved embeddings, respectively, with the best possible value being equal to 1. MR is the Median Rank of the corresponding embedding in the other modality, best possible being equal 1.

Fig 3 (left column) presents the retrieval results for modality pairs trained together for OneProt-5 (top row) and OneProt-4 (bottom row), respectively, showing strong alignment with high R@1 values, especially in sequences paired with structural tokens or graphs. This suggests that the model effectively captures relationships when modalities are explicitly linked during training. Interestingly, while the retrieval performance is good for training modalities, such as sequence paired with structure or graphs, R@1 values remain relatively low for certain modalities, indicating that even with CLIP-style training, perfect synchronization of the encoders is not achieved. This raises the question of whether complete alignment can be attained through pairwise training alone. Furthermore, text alignment performs the worst among the trained modalities, likely due to redundancies in the text data and the inherent complexity of this modality. Fig 3 (right column) shows the results for emergent alignments across unpaired modalities. Note, that OneProt has not seen any of these proteins during training. Generally, the alignment performance is weaker for untrained pairs than trained pairs. Yet, in the worst case, a median rank of 64 is obtained for the task Text→Pocket, which is still considerably lower than the unaligned median rank of 2000 (given the test set size of 4000), providing evidence of an emergent alignment across all tasks.

We also note, that the corresponding alignment values of the OneProt-4 model are higher than those of OneProt-5, suggesting that more training is required to achieve better results for the model with a higher number of modalities. This is supported by the ablation studies shown in S3 Table and can be explained by the growing complexity of the embedding space as the number of modalities increases. Moreover, contrastive loss heavily relies on effective negative sampling. With more modalities, the number of potential negative samples increases significantly, making it harder to distinguish between positive and negative pairs. Future training strategies should aim to enhance the emergent alignments to improve cross-modal adaptability.

### Supervised fine-tuning for downstream tasks

Tables 3 and 4 present the downstream performance of various models across this diverse set of downstream tasks. The evaluation of OneProt-4 and -5 demonstrates its broad applicability in different biological contexts, largely outperforming SaProt with a frozen backbone, also presented in [28], with *p* < 0.01.

**Table 3:** Performance comparison of OneProt, SaProt-LoRa, SaProt, ProTrek, ESM, and OpenFold on five diverse downstream biological protein tasks: ThermoStability (regression), HumanPPI, Metal Ion Binding, DeepLoc Binary (binary classification) and DeepLoc Subcellular (multiclass classification), using Spearman correlation for ThermoStability, accuracy (ACC) and Area Under the Reciever Operating Curve (AUC) for the remaining tasks.

Model	Thermostability	HumanPPI	Metal Ion Binding	DeepLoc	
				Subcellular	Binary
	Spearman’s *ρ*	ACC/AUC%	ACC/AUC%	ACC%	ACC/AUC%
**SaProt-LoRa**	0.724	86.4	75.8	85.6	93.6
**SaProt**	0.702 (0.005)	87.1/92.9 (1.4/2.9)	71.3/76.8 (1.3/1.7)	79.0 (0.4)	91.0/95.6 (0.3/1.4)
**ESM-2**	0.696 (0.005)	86.0/94.2 (1.4/0.6)	67.4/76.2 (1.3/1.7)	81.0 (0.4)	91.4/96.3 (0.4/0.2)
**ESM-3**	0.691 (0.015)	83.6/92.1 (1.8/1.0)	72.7/81.8 (1.9/0.9)	76.3 (0.5)	90.8/95.7 (0.2/0.1)
**ESM-IF**	0.645 (0.006)	78.7/86.0 (1.5/0.6)	69.0/77.7 (1.4/0.6)	61.5 (0.6)	84.7/90.5 (0.5/0.2)
**OpenFold**	0.582 (0.013)	84.4/92.2 (2.3/1.0)	71.7/76.8 (0.7/1.1)	80.0 (0.4)	91.7/96.6 (0.3/0.2)
**ProTrek-35M**	0.638 (0.010)	86.4/94.5 (1.5/0.7)	76.0/94.5 (1.0/0.7)	83.7 (0.3)	93.1/97.8 (0.4/0.1)
**ProTrek-650M**	0.646 (0.009)	90.2/97.0 (1.5/0.5)	75.4/82.3 (3.2/0.4)	90.9 (0.3)	95.3/97.0 (0.2/0.5)
**OneProt-5**	0.673 (0.010)	85.9/93.4 (0.2/0.7)	76.2/82.1 (1.6/1.3)	80.3 (0.2)	92.4/96.3 (0.2/0.2)
**OneProt-4**	0.668 (0.006)	88.8/95.3 (1.7/0.3)	77.3/85.3 (0.5/1.0)	81.7 (0.4)	92.1/96.5 (0.3/0.2)

**Table 4:** Performance comparison of OneProt, SaProt-LoRa, SaProt, ProTrek, ESM, and OpenFold on four multi-label function prediction tasks: Enzyme Commission numbers (EC), Gene Ontology (GO) terms corresponding to Molecular Function (MF), Biological Process (BP), and Cellular Component (CC), using maximum F1-score metric (Fmax) defined by Eq (6).

Model	EC	GO		
		MF	BP	CC
	Fmax	Fmax	Fmax	Fmax
**SaProt-LoRa**	0.884	0.678	0.356	0.414
**SaProt**	0.863 (0.004)	0.623 (0.007)	0.472 (0.004)	0.549 (0.004)
**ESM-2**	0.878 (0.003)	0.645 (0.003)	0.479 (0.003)	0.547 (0.004)
**ESM-3**	0.871 (0.004)	0.643 (0.004)	0.482 (0.003)	0.531 (0.010)
**ESM-IF**	0.896 (0.006)	0.611 (0.005)	0.437 (0.004)	0.488 (0.006)
**OpenFold**	0.888 (0.004)	0.655 (0.005)	0.491 (0.002)	0.548 (0.004)
**ProTrek-35M**	0.846 (0.003)	0.651 (0.002)	0.514 (0.005)	0.583 (0.008)
**ProTrek-650M**	0.876 (0.005)	0.675 (0.006)	0.538 (0.004)	0.617 (0.005)
**OneProt-5**	0.875 (0.005)	0.656 (0.002)	0.492 (0.003)	0.556 (0.005)
**OneProt-4**	0.871 (0.003)	0.656 (0.001)	0.495 (0.003)	0.555 (0.006)

As the results in Table 3 demonstrate, OneProt models achieve competitive performance across all tasks, regardless of the biological differences inherent to each task. Notably, OneProt attains strong results without relying on the more complex fine-tuning in the sequence and structure token modalities or augmenting sequence data with structural tokens, as in Saprot, [28]. Moreover, OneProt outperforms the ESM (*p* < 0.02 for OneProt-5 and *p* < 0.006 for OneProt-4), OpenFold (*p* < 0.046 for OneProt-4) and SaProt (*p* < 0.01) baselines in most tasks, indicating that contrastive learning across different protein encoders effectively aligns them and transfers representational knowledge across modalities. It also delivers comparable (*p* > 0.41 for OneProt-5 on Metal Ion Binding and HumanPPI tasks, two-sided test) to superior (*p* < 0.006 for OneProt-4 on HumanPPI, ThermoStability, Metal Ion Binding, EC and GO-MF tasks; *p* < 0.006 for OneProt-5 on EC, ThermoStability and GO-MF tasks, one-sided test) results to ProTrek-35M on most tasks, and performs similarly (*p* > 0.059 for OneProt-4 on HumanPPI, EC, Metal Ion Binding, and *p* > 0.57 for OneProt-5 on EC and Metal Ion Binding, two-sided test) or better (*p* < 0.002 on ThermoStability) than ProTrek-650M on a number of tasks. We note, however, that in the case of the Metal Ion Binding task, the average performance of ProTrek-650M is lower than that of OneProt models, while the standard deviation is several times higher. Moreover, in terms of AUC, OneProt-4 outperforms ProTrek-650M with *p* < 10^−3^. Other than that, AUC results mainly align with the accuracy scores, but exhibit narrower distributions, indicating greater robustness. Comprehensive *p*-value results from the two-sample Wilcoxon rank-sum test, including both the one-sided test and the two-sided test, are provided in S1–S4 Figs. Moreover, for most downstream tasks, the OneProt-4 and OneProt-5 models yield samples with a lower interquartile range (IQR) compared to the baseline models, as summarized in S8–S11 Tables, indicating robustness of the OneProt results.

The OneProt results are achieved using a substantially smaller pre-training dataset, where modalities are paired only with the sequence modality, more closely reflecting real-world scenarios in which datasets across modalities often differ in size or have limited overlap. This underscores OneProt’s data efficiency and demonstrates, that considerably longer training, as in case of ProTrek-650M (discussed in Materials and methods) does not always lead to significant performance gains. We attribute this to the fact that the additional, graph-based structure and pocket modalities of OneProt provide the information required to achieve good performance on the downstream tasks. Of note, is also that the model OneProt-4, which contains only the GNN encoder for the structure, delivers outstanding performance on the HumanPPI, Metal Ion Binding, GO-MF and -BP tasks and competitive performance across other tasks. Although it is still slightly inferior in performance to OneProt-5 on a number of the other tasks, this suggests that the GNN structure encoder may in general be sufficient for a broad applicability of the model to downstream tasks with datasets of moderate size (for the respective dataset details, please see S1 Table). To test whether matching all modalities improves performance, we trained a version of OneProt-4 on a reduced pre-training set of 300K datapoints, ensuring every datapoint had all four modalities. While this on average increased modality alignment (see S3 Table; OneProt-4 matched), the downstream results were worse than for OneProt-4, S4 Table. Thus, we conclude that modality alignment alone does not determine downstream performance, as the dataset size also plays an important role, even when modalities are not fully matched. Therefore, OneProt is a lightweight, versatile model that efficiently utilizes information stored across modalities without pairing them directly.

### Enzyme function prediction

**Fig 4:**
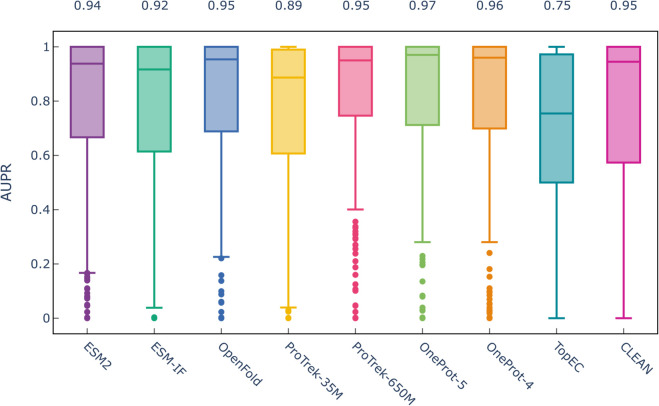
Model performance comparison based on Area Under Precision Recall curve (AUPR) scores for TopEnzyme. Each boxplot shows the AUPR distribution for a method (TopEC, CLEAN, ESM-2, Protrek-35M, ProTrek-650M, OneProt).

Results of the analysis described in the Materials and methods are presented in Fig 4. All embedding-based methods, ESM, ProTrek, and OneProt outperform TopEC (*p* < 10^−6^), while only OneProt-5 significantly outperforms CLEAN (*p* < 0.04). We note, however, that CLEAN results in a higher IQR (0.43) than any one of the embedding-based methods (0.25–0.38), as reported in S6 Table. OneProt-5 achieves significantly higher median AUPR values than the ESM, OpenFold, and ProTrek models (*p* < 0.03). Similarly, OneProt-4 significantly outperforms the ESM and ProTrek-35M models (*p* < 0.02), while delivering a performance comparable to OpenFold and ProTrek-650M, with a tendency toward higher values (*p* > 0.2). Note, on this markedly larger dataset compared to the previous section Supervised fine-tuning for downstream tasks, the OneProt-5 model with the higher number of modalities shows a trend towards better results than OneProt-4 with a tighter IQR. Moreover, as summarized in S6 Table, OneProt-5 also has a low number of outliers (51), corresponding to the values beyond $Q_{1}\;-\;1.5$IQR. The class size distribution of the outliers results in significantly lower values, compared to the overall class size distribution (one-sided Wilcoxon rank sum test *p* < 10^−6^). Therefore, we assume that extending the underrepresented classes could potentially improve the learning and lead to better classification results. We note that CLEAN and TopEC do not have any outliers at all, but they also result in significantly poorer AUPR values than all other models, such that the aforementioned equation yields outlier thresholds with negative values.

The increased performance of ProTrek-650M and OneProt over single-modality methods such as TopEC, CLEAN, and ESM-2 shows the potential of aligned multi-modal protein representations for downstream applications in structural biology.

### Representation learning for evolutionarily related proteins

**Fig 5:**
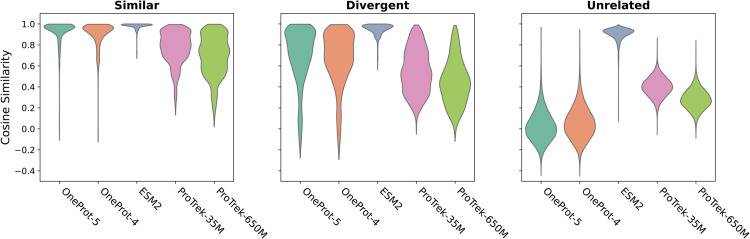
Cosine Similarity distributions for models ESM-2, ProTrek-35M and -650M, OneProt-4 and -5. The plot shows the similarity of a given protein to three groups: the 50 most evolutionarily similar proteins, the 50 most evolutionarily divergent sequences, and 1000 unrelated sequences. While all models partially capture evolutionary relationships, OneProt distinctly separates the three classes, demonstrating its ability to generate meaningful sequence representations.

Fig 5 presents violin plots of cosine similarities across three sequence categories, evolutionarily related similar sequences, evolutionarily related divergent sequences and evolutionarily unrelated sequences, as described in the Materials and methods, for the selected models.

Embeddings from both OneProt models outperform baselines in capturing evolutionary relationships, indicated by a high cosine similarity among related sequences (Fig 5 left and middle), whereas a significantly lower similarity with a large margin for separation is found for unrelated sequences (Fig 5 right, *p* < 10^−16^). We note that ESM-2 and OneProt generally yield high cosine similarity values across related sequence types (*p* < 10^−16^ compared to ProTrek), while ProTrek models show less distinction between dissimilar and unrelated sequences (separation margins <0.2 for ProTrek, >0.6 for OneProt, *p* < 10^−16^). OneProt’s superior capacity to distinguish homologous from non-homologous sequences is likely due to OneProt’s multi-modal training with the InfoNCE loss (Eq (1)). This facilitates the exchange of representational information between encoders, resulting in enriched embeddings that are capable of distinguishing evolutionary relationships. Since OneProt models do not encounter MSA data during training directly, the ability to separate related and unrelated sequences reflects the information shared across encoders.

### ProSPECCTs

We present the AUC results of the analysis as shown in Table 5. The last three columns correspond to the OneProt ablations, which comprise different encoders in addition to the sequence one: structure graph and text encoders only (SG + Text), structure token and text encoders only (ST + Text), structure token, pocket, and text (ST + Text + Pocket). These results showcase the importance of the pocket modality and are discussed in the following section, Ablations.

**Table 5:** AUC Scores for the ProSPECCTs datasets. ST and SG stand for Structure Token and Structure Graph modalities, respectively.

Dataset	Base Models			OneProt Models				
	ESM-2	ProTrek	ProTrek	OneProt-5	OneProt-4	SG + Text	ST + Text	ST + Text
		650M	35M					+Pocket
DS1	1.000	1.000	1.000	1.000	1.000	1.000	1.000	1.000
DS1.2	1.000	1.000	1.000	1.000	1.000	1.000	1.000	1.000
DS2	1.000	1.000	1.000	1.000	1.000	0.998	0.998	1.000
DS3	0.878	0.577	0.578	0.881	0.874	0.801	0.868	**0.899**
DS4	0.866	0.576	0.581	0.859	0.850	0.779	0.877	**0.880**
DS5 & 5.2	0.529	0.578	0.585	0.646	0.632	0.639	**0.653**	0.650
DS6	0.520	0.593	0.531	**0.621**	0.551	0.527	0.602	0.587
DS6.2	0.520	0.592	0.531	**0.620**	0.550	0.527	0.602	0.587
DS7	0.654	0.782	0.703	0.843	**0.848**	0.834	0.840	0.843

Remarkably, even without task-specific fine-tuning, OneProt models tend to outperform all others across all datasets. Notably, for DS5-7, all OneProt models achieve substantially higher AUC values compared to the baselines.

Given that OneProt-5 exhibited consistently good performance across all ProSPECCTs datasets, surpassing ESM-2 everywhere but DS4, we have also investigated the predictive power of its structure, pocket, and joint concatenated embeddings of OneProt-5 (see S6 Table). Yet, the OneProt-5 sequence encoder typically remained superior. Namely, despite the pocket encoder being trained on binding site data, the sequence encoder in OneProt-5 outperforms it, likely due to its larger pre-training dataset providing a broader understanding of protein properties. The one exception is DS7, where pocket embeddings and combined structure–sequence embeddings surpass the OneProt sequence embeddings. We attribute this to DS7’s focus on recovering binding-site similarity across highly divergent proteins, making structural and pocket features especially discriminative in that setting.

By integrating sequence-level knowledge with binding site-specific data during pre-training, OneProt demonstrates an enhanced ability to distinguish subtle chemical variations, as systematically evaluated in diverse contexts using the ProSPECCTs benchmark datasets. Moreover, it showcases the added benefit of multi-modal training atop of the pre-trained ESM-2.

OneProt’s representations appear to better understand the subtle molecular recognition distinctions between protein-ligand complexes, particularly in datasets where proteins bind to the same ligand but exhibit slight structural variations. This observation is especially pronounced in DS3 and DS4, where protein structures are generally very similar, but nuanced changes in chemical interactions are present.

### Ablations

We discuss here the most noteworthy ablations of OneProt, while the data for the rest, including the Figures, are provided in S3-S7 Tables, S1-S5 Figs.

**Fig 6:**
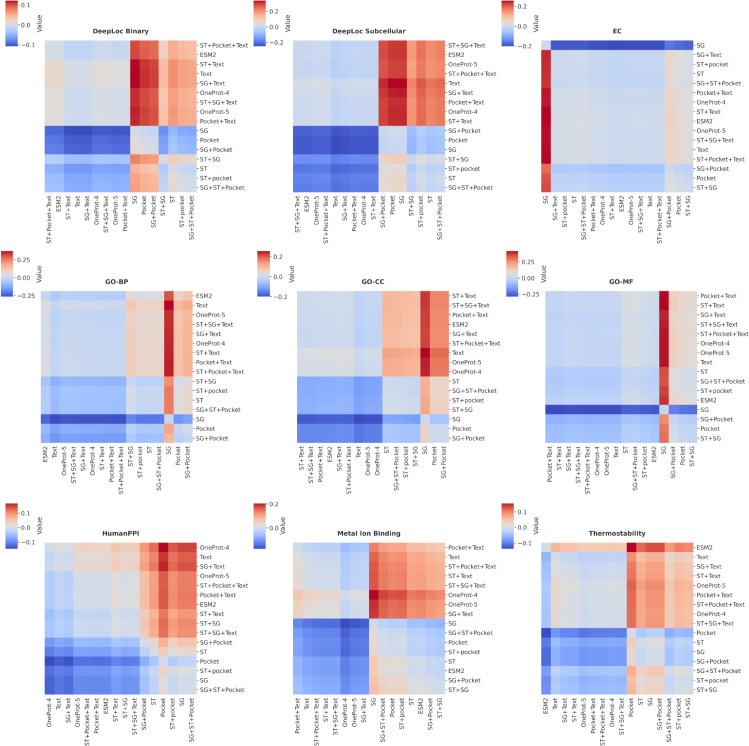
Normalized performance drops $\Delta_{x,y}=\frac{Perf_{y}-Perf_{x}}{Perf_{x}}$. The heatmaps visualize the Eq (7) applied to the OneProt ablations, where *x* is the model on the horizontal axis, while *y* is the model on the vertical axis. Colours in the shades of blue correspond to negative values (model on the horizontal axis outperforms model on the vertical axis), and colours in the shades of red correspond to positive ones.

Ablations corresponding to section Supervised fine-tuning for downstream tasks are summarized in Tables 6 and S4 and Fig 6. The latter presents a heatmap, where negative $\Delta_{x,y}$, Eq (7), indicating inferiority of the model on the vertical axis *y* compared to model on the horizontal axis *x*, are shown in shades of blue, and positive $\Delta_{x,y}$ in shades of red. We note that the model comprising only sequence and text encoders exhibits outstanding performance on DeepLoc tasks. We attribute the high accuracy on the DeepLoc2 downstream task to the alignment between sequence and text encoders: all models, where the sequence-text alignment was higher than 0.22, exhibited an accuracy above 92.7%, as shown in Fig 6 (DeepLoc Binary), where three darkest vertical blue lines mark the text, text and structure graph (Text + SG), text and structure token (Text + ST) models. Moreover, the corresponding three models also exhibited statistically comparable performance with ProTrek-35M (*p* > 0.09, two-sided test). This, however, does not guarantee high performance on the DeepLoc10 task, where a relatively high 82.2% accuracy is achieved also by the encoder comprising sequence, structure graph, and text (SG + Text). The latter may have to do with the fact that the structure modality in that case is almost perfectly aligned with the sequence (R@1>0.95, R@10=1.0), as well as with the satisfactory emergent structure graph-text alignment (R@1≈0.1, R@10≈0.4), while the text-sequence alignment remains at a high level (S3 Table). Clustering in Fig 6 indicates the best performing models as those comprising the text encoder (Text), with Text and SG + Text corresponding to the darkest vertical blue lines on the heatmap.

**Table 6:** Downstream results of the selected ablations on the datasets from [28]. Abbreviations as in Tables 3–5.

Model	Thermostability	HumanPPI	Metal Ion Binding	EC	GO			DeepLoc	
					MF	BP	CC	Subcellular	Binary
	Spearman’s *ρ*	ACC/AUC%	ACC/AUC%	Fmax	Fmax	Fmax	Fmax	ACC%	ACC/AUC%
**Text**	0.656 (0.005)	87.5/94.3 (1.4/0.5)	74.0/81.3 (2.2/2.0)	0.876 (0.003)	0.656 (0.004)	**0.503** (0.002)	**0.561** (0.005)	**83.0** (0.4)	92.9/97.3 (0.2/0.1)
**Text + SG**	0.664 (0.015)	**88.1/96.4** (1.3/1.3)	**75.9/85.1** (0.8/0.4)	0.866 (0.003)	0.651 (0.004)	0.495 (0.004)	0.545 (0.004)	82.2 (0.3)	**92.9/96.9** (0.4/0.3)
**Text + ST**	0.666 (0.014)	84.5/92.7 (1.2/1.1)	74.7/81.8 (1.1/1.7)	**0.877** (0.001)	**0.661** (0.002)	0.496 (0.003)	0.549 (0.007)	81.6 (0.4)	92.8/96.9 (0.3/0.1)
**Text + ST + Pocket**	**0.670** (0.006)	85.9/93.9 (1.6/0.5)	74.5/81.8 (1.0/0.5)	0.876 (0.004)	0.657 (0.005)	0.497 (0.004)	0.546 (0.004)	80.5 (0.5)	91.3/96.3 (0.3/0.2)

We observe that combining the structure graph and pocket encoders significantly improves performance on the HumanPPI and Metal Ion Binding tasks (*p* < 0.04 and *p* < 0.003, respectively, comparing SG + Pocket + Text to a Text-only model, one-sided tests). This improvement is likely due to the complementary information that these modalities provide about binding and interaction sites. Replacing the structure graph with the structure token (ST) encoder, however, reduces performance (*p* < 10^−3^ comparing SG + Pocket + Text with ST + Pocket + Text, one-sided tests), as the token encoder tends to capture global structural similarity rather than local spatial relationships or graph-like geometries. These aspects are critical for describing interface regions in the HumanPPI task and ensuring local geometric precision in Metal Ion Binding sites. The superiority of the discussed models is further evident in Fig 6, where the OneProt-4 model is marked by vertical lines in the darkest blue.

For the EC number prediction task, however, we observe that incorporating the ST encoder alongside the text encoder leads to superior performance compared to using the GNN encoder for structure, which reduces the score for this task (*p* < 0.01 when comparing any model incorporating both ST and Text with models that include SG but omit ST). On this dataset, the information captured by the structure token encoder proves more valuable than the geometric details encoded by the GNN.

In the TopEnzyme task, which also focuses on EC number prediction, the inclusion of structure token and text encoders in the OneProt models results in AUPR medians of at least 0.95, with a fairly tight distribution of AUPR values (IQR 0.27-0.3) (S5 Fig and S6 Table). Yet, it is the pocket encoder, which consistently contributes to performance improvements across models. The improvement is statistically significant when comparing ST with ST + Pocket, and ST + SG with ST + SG+Pocket (*p* < 0.02). For other model pairs, the pocket encoder generally tends to reduce variability by narrowing the IQR and/or decreasing the number of outliers, while maintaining or slightly improving the median AUPR. An exception is observed in the OneProt-5 model, where the number of outliers did not decrease relative to the ST + SG + Text model, but the median AUPR increased (0.97 vs. 0.95). This emphasizes the importance of adding localized structural information, thereby, confirming the findings of [16]. We note that although both EC and TopEnzyme tasks are related to EC numbers predictions, the key encoders for good performance differ, due to the differences in dataset composition. While the TopEnzyme benchmark was specifically designed to better capture EC number differences that depend on active-site geometry, therefore favoring models with pocket-based encoders, the EC task uses broader EC class distinctions and is rich in evolutionary or sequence motif signals. This provides a rationale for why models using ST encoders, which are known to map sequence context well into token space, perform more strongly on the EC task than the TopEnzyme task.

For predicting evolutionarily-related sequences, the model with only the text encoder appears to fail completely, likely because the text encoder focuses on function-to-sequence associations rather than direct sequence comparisons, S6 Fig. Models relying solely on GNN encoders generally capture evolutionary relationships but exhibit higher variance due to their sensitivity to small structural perturbations. Notably, the combination of GNN structure, pocket, and text encoders achieves superior performance, highlighting the complementary nature of these encoders.

Finally, in the ProSPECCTs task, we note again the importance of the pocket encoder for the DS3-4 datasets, both alone and in combination with other modalities, Tables 5 and S5. The predictions on datasets DS6-6.2, on the contrary, significantly benefit from the inclusion of the structure token encoder. The reason, again, lies in the way the datasets were constructed: DS3 and 4 are datasets with similar protein structures but different physicochemical binding sites and binding shape properties, respectively, which can be captured by node and edge features of a GNN pocket encoder; DS6-6.2 are about distant relationships between protein binding sites with identical ligands that have similar environments, which are tasks more suited for the structure token encoder, capturing global structure-based similarities or environment-level context, whereas GNN encoders are more focused on the local information.

In summary, while certain encoders are well-suited to specific downstream tasks, no single encoder model consistently delivers superior performance across the full spectrum of tasks discussed. Moreover, adding certain modalities to a model can degrade performance if the encoder is not well-suited for the requirements of the downstream task. Leveraging the complementary strengths of multiple encoders, while keeping an eye on the modality alignment, is often essential for addressing the diverse and complex nature of protein-related problems. Notably, we highlight the critical role of the GNN structure and pocket encoders, features absent in the otherwise conceptually similar ProTrek models, compared to relying solely on the structure token encoder.

## Discussion

This work contributes significantly to the development of multi-modal protein foundation models by implementing a comprehensive framework that extends the ImageBind concept into the protein domain. In addition, we present a lightweight fine-tuning scheme for downstream tasks, designed to align more closely with real-world requirements, enabling efficient adaptation without the need for extensive computational resources or large-scale datasets. The modular and extendable codebase allows easy integration of new modalities using pre-trained encoders, providing a flexible interface for a multitude of downstream tasks, thereby making it highly adaptable and versatile. This design supports efficient ablation studies to assess the impact of specific encoders or their combinations. Based on these insights, we identified two OneProt models, utilizing 4 and 5 encoders, that consistently delivered strong performance across all downstream tasks discussed. More specifically, the present study demonstrates emergent alignment between modalities not paired during training, underscoring the framework’s capability for cross-modal alignment. Our models yield competitive performance across downstream tasks, including enzyme function prediction, the ProSPECCTs benchmarking initiative, and standard tasks proposed in [28], often achieving state-of-the-art or better results.

Consistently high R@1 values in Fig 3 demonstrate OneProt’s strong alignment accuracy between trained modality pairs. Despite lacking established benchmarks to quantitatively contextualize these rank metrics, achieving median ranks below 100 (often below 10) across 4,000 samples indicates effective latent space alignment and, thereby, supports the validity of our multi-modal approach. Furthermore, retrieval tasks confirm OneProt’s precise cross-modal alignment, underscoring the robustness and adaptability of our framework in integrating diverse protein data and mirroring ImageBind’s achievements [12], now in the protein domain.

By generating embeddings for simple MLP classifiers, OneProt highlights the effectiveness of aligned multi-modal protein representations for complex classification tasks in structural biology. As such, OneProt leads to a strong performance on the challenging TopEnzyme task, achieving a median 0.97 AUPR in enzyme function prediction. Moreover, the evaluation on ProSPECCTs benchmark datasets for binding sites analysis demonstrates that our model’s sequence embeddings perform consistently better than ESM-2 and ProTrek across all datasets, with the exception of DS4, where OneProt performs comparably to ESM-2, while still beating ProTrek by a pronounced margin, indicating that the additional pocket and structure encoders of OneProt contribute to the results. Finally, we demonstrated that OneProt’s contrastive learning approach captures evolutionary relationships with greater fidelity than ESM-2 and ProTrek, as evidenced by the lower similarity scores for unrelated sequences. This highlights the efficacy of our multi-modal CLIP training in generating biologically meaningful protein representations.

Importantly, OneProt should not be seen as a direct competitor to more complex multi-modal models, such as SaProt-LoRa [28] or ProTrek-650M [32] as shown in section Results, where these models retain higher performance in some benchmarks. Notably, the inclusion of a GNN encoder in OneProt equips it to capture relational dependencies in protein sequences. This design enables OneProt to deliver competitive performance compared to these substantially larger models on tasks including HumanPPI, Metal Ion Binding, TopEnzyme, ProSPECCTs, and evolutionary relatedness, despite being trained on much smaller datasets, with significantly fewer optimizer steps, and using a more memory-efficient fine-tuning scheme compared to SaProt. We also observed that ESM-3 [20], when applied to the Saprot [28] datasets, did not demonstrate notable improvements in predictive performance. Additionally, it required substantially higher memory during inference and showed reduced effectiveness on the other tasks when sequence length cut-offs were applied. As a result, we opted not to include it in our analysis beyond the section Supervised fine-tuning for downstream tasks to maintain focus on the models that are more computationally efficient and better suited for the specific constraints of our downstream tasks. With this in mind, we note that OneProt demonstrates how aligning specialized encoders can create a cohesive multi-modal system that transfers information effectively across modalities, remaining flexible to incomplete or heterogeneous data while requiring only moderate computational resources. Additionally, the multi-modal training approach has generated representations that robustly preserve evolutionary biological structures, providing a foundation for capturing the complex relationships inherent in protein data across diverse tasks. The models’ ablations described in section Ablations highlight and make understandable the contributions of the specific encoders to downstream tasks. That said, we note that as we add more modalities, our current simple parallelization scheme may become less efficient due to larger memory requirements. In future work, one could adopt more GPU-efficient strategies, which could also potentially handle longer protein sequences. Alternatively, to overcome the memory issues, one could focus on a curated subset of the most relevant modalities, while still using the existing framework.

Beyond current applications, OneProt provides foundational work for expanding multi-modal protein representation models into broader biological tasks, such as multi-target drug design or protein-protein interaction prediction in complex diseases, by integrating OneProt embeddings into diffusion or large language models. This flexibility highlights OneProt’s role in integrative biological research and computational drug discovery, where multi-modal, data-rich environments are essential for achieving translational success. Future research can explore additional modalities, e.g., multiple conformations for a protein and structures of protein-small molecule complexes, protein representations enriched with protein-protein interaction information, using recent encoders [62], [63], or protein-nucleic acid complexes, acknowledging that such data may not be universally available for all proteins. In addition, information on proteins under different environmental, experimental, or physiological conditions could be added, which may further refine OneProt’s capacity to analyze proteins under various physicochemical conditions. A deeper analysis of the evolutionary relatedness between embeddings could be conducted, quantifying contributions of structural or protein-protein interaction information, and of the transfer of information from different modalities to the sequence embeddings. Moreover, extending the current contrastive loss framework could allow moving beyond treating only pairs of modalities as positives. Instead, one could emphasize the clustering of proteins that participate in, e.g., the same disease pathway, and incorporate such relational information into the model via the encoders described in [64].

## Supporting information

S1 Text Pre-training. S1.1 Training details and S1.2 Metrics.
(PDF)

S2 Text Supervised Fine-tuning Downstream Tasks. (PDF)

S3 Text Dataset details. (PDF)

S4 Text Abbreviations glossary. (PDF)

S1 Table Overview of supervised Downstream Datasets from [28] and [56]. The data splits follow those provided in the original studies and reflect the same clustering strategy used therein. For multi-label classification tasks, the number of classes is listed in parentheses.
(PDF)

S2 Table Overview of ProSPECCTs Datasets. (PDF)

S3 Table Modality alignments for selected ablations in terms of R@1 and (in parentheses) R@10. ST. corresponds to the Structure Token modality, SG corresponds to the Structure Graph modality, ‘+’ indicates the combination of multiple modalities.
(PDF)

S4 Table Downstream results for the ablations not included in the main text on the datasets from [28]. The tasks comprise ThermoStability (regression) evaluated using Spearman correlation, HumanPPI, Metal Ion Binding, DeepLoc (binary) and DeepLoc Subcellular (multiclass classification), evaluated using accuracy (ACC) and Area Under the Receiver Operating Curve (AUC), Enzyme Commision numbers (EC), Gene Ontology (GO) terms corresponding to Molecular Function (MF), Biological Process (BP) and Cellular Component (CC) evaluated using maximum F1-score metric (Fmax) defined by Eq (6) of the main text. ST corresponds to the Structure Token modality, SG corresponds to the Structure Graph modality, ‘+’ indicates the combination of multiple modalities.
(PDF)

S5 Table Receiver Operating Characteristic Area Under the Curve (AUC) scores for the ProSPECCTs datasets for the ablations of OneProt not included in the main text. ST corresponds to the Structure Token modality, SG corresponds to the Structure Graph modality, ‘+’ indicates the combination of multiple modalities.
(PDF)

S6 Table Receiver Operating Characteristic Area Under the Curve (AUC) scores for the ProSPECCTs datasets using alternatives from sequence embeddings from OneProt-5. For the last two columns, concatenated embeddings are used for prediction.
(PDF)

S7 Table Numerical values corresponding to boxplots in Figs 4 and S4. Q1 and Q3 correspond to the first and third quartiles of the data, respectively. Column outliers correspond to the count of values below Q1-1.5×IQR.
(PDF)

S8 Table Table of ranges (Min, Max), 0.25 (Q1), 0.5 (Median), 0.75 (Q3), Inter Quantile Range (IQR = Q3 - Q1) for metrics of different models on DeepLoc2, DeepLoc10 (accuracy), EC (Fmax) tasks. (PDF)

S9 Table Table of ranges (Min, Max), 0.25 (Q1), 0.5 (Median), 0.75 (Q3), Inter Quantile Range (IQR = Q3 - Q1) for metrics of different models on GO-BP, GO-CC, GO-MF (Fmax) tasks. (PDF)

S10 TableTable of ranges (Min, Max), 0.25 (Q1), 0.5 (Median), 0.75 (Q3), Inter Quantile Range (IQR = Q3 - Q1) for metrics of different models on HumanPPI, Metal Ion Binding (accuracy), ThermoStability (Spearman’s ρ) tasks.(PDF)

S11 Table Table of ranges (Min, Max), 0.25 (Q1), 0.5 (Median), 0.75 (Q3), Inter Quantile Range (IQR = Q3 - Q1) for AUC of different models on binary classification tasks: DeepLoc2, HumanPPI, Metal IonBinding) tasks. (PDF)

S1 Fig Heatmaps of *p*-values according to one-sided Wilcoxon rank-sum test. The alternative hypothesis of OneProt models (vertical axis) outperforming baseline models (horizontal axis) according to metric values from Tables 3 and 4, where for binary classification accuracy was compared. Striped pattern stands for the values *p* < 0.05, when the null hypothesis of OneProt being non-superior was rejected.
(PDF)

S2 FigHeatmaps of *p*-values according to two-sided Wilcoxon rank-sum test.The alternative hypothesis of OneProt models (vertical axis) performing differently from baseline models (horizontal axis) according to metric values from Tables 3 and 4, where for binary classification accuracy was compared. Striped pattern stands for the values p≥0.05, when the null hypothesis of OneProt being the same as the baseline was not rejected.(PDF)

S3 FigHeatmaps of *p*-values for Area Under Receiver Operating Characteristic curve metrics.One-sided Wilcoxon rank-sum test with the alternative hypothesis of OneProt (vertical axis) outperforming baseline models (horizontal axis), striped pattern corresponding to values *p* < 0.05 (upper panel). Two-sided Wilcoxon rank-sum test with the alternative hypothesis of OneProt performing differently than baseline models, striped pattern corresponding to values p≥0.05 (middle panel). One-sided Wilcoxon rank-sum test for OneProt ablations with the alternative hypothesis of the models on the vertical axis outperforming the models on the horizontal axis (bottom panel). Striped pattern stands for the values *p* < 0.05.(PDF)

S4 Fig Heatmaps of *p*-values according to one-sided Wilcoxon rank-sum test for OneProt ablations. The alternative hypothesis of the models on the vertical axis outperforming the models on the horizontal according to metric values from Tables 3 and 4, where for binary classification accuracy was compared. Striped pattern stands for the values *p* < 0.05, when the null hypothesis of model on the vertical axis being non-superior was rejected.
(PDF)

S5 Fig Performance comparison of OneProt ablations on the TopEnzyme dataset using boxplots of the Area Under Precision Recall curve (AUPR) distributions. ST corresponds to the Structure Token modality, SG corresponds to the Structure Graph modality, ‘+’ indicates the combination of multiple modalities.
(PDF)

S6 Fig Cosine Similarity distributions for OneProt ablations. The plot shows the similarity of a given protein to three groups: the 50 most evolutionarily similar proteins, the 50 most evolutionarily divergent sequences, and 1000 unrelated sequences. ST corresponds to the Structure Token modality, SG corresponds to the Structure Graph modality, ‘+’ indicates the combination of multiple modalities.
(PDF)
