# Supplementary material for: OneProt: Towards multi-modal protein foundation models via latent space alignment of sequence, structure, binding sites and text encoders
Source: PLoS Comput Biol. 2025 Nov 13;21(11):e1013679. doi: 10.1371/journal.pcbi.1013679 (PMC12614600; doi:10.1371/journal.pcbi.1013679)
Supplement: S1 Text — S1.1 Training details and S1.2 Metrics. (PDF) [file pcbi.1013679.s001.pdf]

S1 Pre-training

S1.1 Training details

We used the AdamW optimizer [2] with a constant learning rate schedule 0.001, the temperature parameter τ in the loss function (Equations (1)-(2) of the main text) set to 1. The batch size for each of the modalities was 32.

During training, data from different modalities were combined using PyTorch Lightning's CombinedDataloader in min_size mode, ensuring that each epoch's dataset size matched the smallest modality. Therefore, at the beginning of each epoch larger modalities were subsampled accordingly. For validation, sequential mode was used to enable exhaustive evaluation on the validation dataset.

The models were run on JUWELS BOOSTER [1] on 16 nodes with each compute node comprising four NVIDIA A100 GPUs for 33000 optimizer steps. Parallelization was implemented using PyTorch's Distributed Data Parallel scheme, which loads the model on each GPU, thereby minimizing inter-node overhead and resulting in faster training performance. Because the model is loaded onto each GPU, it is light enough to run on a single GPU, and we used 64 GPUs only to accelerate training. The seeds were fixed during pre-training and are available in the corresponding config files in the github repository.

S1.2 Metrics

To show the model's retrieval capabilities evolving over training, we report the median rank across two types of retrieval tasks. For retrieval between modalities that were trained together (such as sequence paired with another modality), we calculate the median rank of cosine similarities of the same protein representations, one of which includes the sequence representation, and average them out.

For emergent retrieval tasks, which involve pairing modalities not directly trained together, we evaluate the ability of the model to generalize to untrained modality pairs. Similarly to the previous case, we measure the median rank of cosine similarity between representations of the same protein across each pair of modalities, not including the sequence one, and average over the respective emergent retrieval tasks. This approach provides a robust indication of how well the model captures transferable features between previously unlinked modalities.

References

[1] Stefan Kesselheim, Andreas Herten, Kai Krajsek, Jan Ebert, Jenia Jitsev, Mehdi Cherti, Michael Langguth, Bing Gong, Scarlet Stadtler, Amirpasha Mozaffari, Gabriele Cavallaro, Rocco Sedona, Alexander Schug, Alexandre Strube, Roshni Kamath, Martin G. Schultz, Morris Riedel, and Thomas Lippert. Juwels booster – a supercomputer for large-scale ai research. In Heike Jagode, Hartwig Anzt, Hatem Ltaief, and Piotr Luszczek, editors,

High Performance Computing, pages 453–468, Cham, 2021. Springer International Publishing.

[2] Ilya Loshchilov and Frank Hutter. Decoupled weight decay regularization, 2019.
