# Supplementary material for: OneProt: Towards multi-modal protein foundation models via latent space alignment of sequence, structure, binding sites and text encoders
Source: PLoS Comput Biol. 2025 Nov 13;21(11):e1013679. doi: 10.1371/journal.pcbi.1013679 (PMC12614600; doi:10.1371/journal.pcbi.1013679)
Supplement: S2 Text — (PDF) [file pcbi.1013679.s002.pdf]

S2 Supervised fine-tuning Downstream Tasks

For our supervised downstream tasks, we initially generate embeddings using the sequence reference and projection head of our OneProt model. These embeddings are created for all proteins in the datasets from [1] and [2], covering a total of ten datasets. Subsequently, we train a Multi-Layer Perceptron (MLP) on these embeddings. We conducted a comprehensive hyperparameter sweep, exploring a variety of settings:

- **Learning Rates:** 0.001, 0.01
- **Batch Sizes:** 32, 64
- **Maximum Epochs:** 50
- **Hidden Dimensions:** [256], [512, 256]
- **Dropout Rates:** 0.1, 0.25
- **Normalization:** Batch normalization (enabled/disabled), Layer normalization (enabled/disabled)
- **Activation Functions:** "relu", "gelu"
- **Residual Connections:** enabled, disabled

These hyperparameter combinations provided a robust evaluation of the MLP model across various biological and biochemical tasks, allowing us to fine-tune the model's performance for each specific task while offering the flexibility to experiment with different models efficiently. The MLP-based approach is simpler and considerably faster, as it eliminates the need for fine-tuning the entire protein encoder model using LoRA, as done for the Saprot[1]. This allows for faster iteration and evaluation cycles, as we can directly use precomputed embeddings and quickly train the MLP with various configurations. Moreover, this setup is highly flexible, enabling the easy integration of other supervised learning models, such as logistic regression, random forests, gradient-boosted trees, and support vector machines. This adaptability makes it straightforward to experiment with multiple models and identify the best-performing one for each specific task.

References

[1] Jin Su, Chenchen Han, Yuyang Zhou, Junjie Shan, Xibin Zhou, and Fajie Yuan. Saprot: Protein language modeling with structure-aware vocabulary. *bioRxiv*, 2023.

[2] Karel J van der Weg and Holger Gohlke. Topenzyme: a framework and database for structural coverage of the functional enzyme space. *Bioinformatics*, 39(3), 2023.
