# Supplementary material for: OneProt: Towards multi-modal protein foundation models via latent space alignment of sequence, structure, binding sites and text encoders
Source: PLoS Comput Biol. 2025 Nov 13;21(11):e1013679. doi: 10.1371/journal.pcbi.1013679 (PMC12614600; doi:10.1371/journal.pcbi.1013679)
Supplement: S3 Text — (PDF) [file pcbi.1013679.s003.pdf]

S3 Dataset Details

To create the full training dataset, we combined data from multiple sources. We started with the OpenProteinSet [1] as a basis for training due to the high availability of MSA data. This database contains structures, sequences, and MSAs for proteins from the PDB [3] and proteins from UniClust30 [5]. We extend this database with proteins from UniProtKB/Swiss-Prot [2] as these proteins are experimentally studied and usually have information across multiple modalities available. Using MMseqs2 [6], we clustered the obtained sequences from OpenFold and Swiss-Prot with a sequence identity cut-off of 50%, such that each cluster represents a homologous cluster in the protein fold space [8]. We align the training, validation, and test split along these sequence clusters. For each cluster representative and member, using the sequence, we find the structure from the AlphaFold2DB [9], the MSA from the OpenProteinSet, and the binding pocket with P2Rank [4] including a 100 closest residues to the predicted binding site, as in [7]. As we could not find a binding pocket for each protein, fewer data points for these modalities are available.

References

[1] Gustaf Ahdritz, Nazim Bouatta, Sachin Kadyan, Lukas Jarosch, Daniel Berenberg, Ian Fisk, Andrew M. Watkins, Stephen Ra, Richard Bonneau, and Mohammed AlQuraishi. Openproteinset: Training data for structural biology at scale, 2023.

[2] Emmanuel Boutet, Damien Lieberherr, Michael Tognolli, Michel Schneider, and Amos Bairoch. *UniProtKB/Swiss-Prot*, pages 89–112. Humana Press, Totowa, NJ, 2007.

[3] Stephen K Burley, Charmi Bhikadiya, Chunxiao Bi, Sebastian Bittrich, Henry Chao, Li Chen, Paul A Craig, Gregg V Crichlow, Kenneth Dalenberg, Jose M Duarte, et al. Rcsb protein data bank (rcsb. org): delivery of experimentally-determined pdb structures alongside one million computed structure models of proteins from artificial intelligence/machine learning. *Nucleic acids research*, 51(D1):D488–D508, 2023.

[4] R. Krivák and D. Hoksza. P2rank: machine learning based tool for rapid and accurate prediction of ligand binding sites from protein structure. *J Cheminform*, 10, 2018.

[5] Mirdita M., von den Driesch L., Galiez C., Martin M. J., Söding J., and Steinegger M. Uniclust databases of clustered and deeply annotated protein sequences and alignment. *Nucleic Acids Res*, 2016.

[6] M. Steinegger and J. Söding. Mmseqs2 enables sensitive protein sequence searching for the analysis of massive data sets. *Nat Biotechnol*, 35:1026–1028, 2017.

[7] Karel van der Weg, Erinc Merdivan, Marie Piraud, and Holger Gohlke. Topec: Improved classification of enzyme function by a localized 3d protein descriptor and 3d graph neural networks. *bioRxiv*, 2024.

[8] Karel J van der Weg and Holger Gohlke. Topenzyme: a framework and database for structural coverage of the functional enzyme space. *Bioinformatics*, 39(3), 2023.

[9] Mihaly Varadi, Stephen Anyango, Mandar Deshpande, Sreenath Nair, Cindy Natassia, Galabina Yordanova, David Yuan, Oana Stroe, Gemma Wood, Agata Laydon, Augustin Žídek, Tim Green, Kathryn Tunyasuvunakool, Stig Petersen, John Jumper, Ellen Clancy, Richard Green, Ankur Vora, Mira Lutfi, Michael Figurnov, Andrew Cowie, Nicole Hobbs, Pushmeet Kohli, Gerard Kleywegt, Ewan Birney, Demis Hassabis, and Sameer Velankar. AlphaFold Protein Structure Database: massively expanding the structural coverage of protein-sequence space with high-accuracy models. *Nucleic Acids Research*, 50(D1):D439–D444, 11 2021.
