# Supplementary material for: OneProt: Towards multi-modal protein foundation models via latent space alignment of sequence, structure, binding sites and text encoders
Source: PLoS Comput Biol. 2025 Nov 13;21(11):e1013679. doi: 10.1371/journal.pcbi.1013679 (PMC12614600; doi:10.1371/journal.pcbi.1013679)
Supplement: S4 Text — (PDF) [file pcbi.1013679.s004.pdf]

S4 Abbreviations glossary

AI Artificial Intelligence

AUC Area Under Receiver Operatoing Characteristic curve

AUPR Area Under Precision-Recall curve

BP Biological Process

CC Cellular Component

CLIP Contrastive Language-Image Pre-training

DDP Distributed Data Parallel

EC Enzyme Commission

ESM Evolutionary Scale Modeling

IF Inverse Fold

IQR Inter-Quantile Range

GNN Graph Neural Network

GO Gene Ontology

GPT Generative Pre-trained Transformer

GPU Graphics Processing Unit

HumanPPI Human Protein Protein Interactions

InfoNCE Information Noise-Contrastive Estimation

LoRA Low-rank Adaptation

MF Molecular Function

MLM Masked Language Modeling

MLP Multi-Layer Perceptron

MR Median Rank

MSA Multiple Sequence Alignment

NMR Nuclear Magnetic Resonance

ProSPECCTs Protein Site Pairs for the Evaluation of Cavity Comparison Tools

SG Structure Graph

ST Structure Token
