# Supplementary material for: OneProt: Towards multi-modal protein foundation models via latent space alignment of sequence, structure, binding sites and text encoders
Source: PLoS Comput Biol. 2025 Nov 13;21(11):e1013679. doi: 10.1371/journal.pcbi.1013679 (PMC12614600; doi:10.1371/journal.pcbi.1013679)
Supplement: S1 Table — The data splits follow those provided in the original studies and reflect the same clustering strategy used therein. For multi-label classification tasks, the number of classes is listed in parentheses. (PDF) [file pcbi.1013679.s005.pdf]

Table S1: Overview of supervised Downstream Datasets from [5] and [6]. The data splits follow those provided in the original studies and reflect the same clustering strategy used therein. For multi-label classification tasks the number of classes is listed in parentheses.

**Dataset**	**Type**	**Category**	**Evaluation Metric**	**Train**	**Valid**	**Test**
Thermostability [2]	Regression	Human-Cell	Spearman's ρ	5056	639	1336
MetalIonBinding [4]	Binary classification	-	Acc	4247	662	665
DeepLoc [1]	Binary classification	Binary	Acc	5477	1336	1731
	Multi-class classification (10)	Subcellular	Acc	8747	2191	2747
HumanPPI [7]	Binary classification	-	Acc	26319	234	180
EC [3]	Multi-label classification (585)	-	Fmax	13089	1465	1604
GO [3]	Multi-label classification (1943 / 489 / 320)	BP / MF / CC	Fmax	26224	2904	3350
TopEnzyme [6]	Multi-label classification (826)	-	Fmax	224531	15149	17488

References

[1] José Juan Almagro Armenteros, Casper Kaae Sønderby, Søren Kaae Sønderby, Henrik Nielsen, and Ole Winther. DeepLoc: prediction of protein subcellular localization using deep learning. *Bioinformatics*, 33(20):3387–3395, October 2017.

[2] Christian Dallago, Jody Mou, Kadina E. Johnston, Bruce J. Wittmann, Nicholas Bhattacharya, Samuel Goldman, Ali Madani, and Kevin K. Yang. FLIP: Benchmark tasks in fitness landscape inference for proteins. *bioRxiv*, 2021.

[3] Vladimir Gligorijević, P. Douglas Renfrew, Tomasz Kościołek, Julia Koehler Leman, Daniel Berenberg, Tommi Vatanen, Chris Chandler, Bryn C. Taylor, Ian M. Fisk, Hera Vlamakis, Ramnik J. Xavier, Rob Knight, Kyunghyun Cho, and Richard Bonneau. Structure-based protein function prediction using graph convolutional networks. *Nature Communications*, 12(1):3168, May 2021.

[4] Mingyang Hu, Fajie Yuan, Kevin Yang, Fusong Ju, Jin Su, Hui Wang, Fei Yang, and Qiuyang Ding. Exploring evolution-aware and -free protein language models as protein function predictors. In Sanmi Koyejo, Shakir Mohamed, Alekh Agarwal, Danielle Belgrave, Kyunghyun Cho, and Alice Oh, editors, *Advances in Neural Information Processing Systems*, volume 35, pages 38873–38884. Curran Associates, Inc., 2022.

[5] Jin Su, Chenchen Han, Yuyang Zhou, Junjie Shan, Xibin Zhou, and Fajie Yuan. Saprot: Protein language modeling with structure-aware vocabulary. *bioRxiv*, 2023.

[6] Karel J van der Weg and Holger Gohlke. Topenzyme: a framework and database for structural coverage of the functional enzyme space. *Bioinformatics*, 39(3), 2023.

[7] Minghao Xu, Zuobai Zhang, Jiarui Lu, Zhaocheng Zhu, Yangtian Zhang, Chang Ma, Runcheng Liu, and Jian Tang. PEER: A comprehensive and multi-task benchmark for protein sequence understanding. *arXiv preprint arXiv:2206.02096*, 2022.
