# Supplementary material for: OneProt: Towards multi-modal protein foundation models via latent space alignment of sequence, structure, binding sites and text encoders
Source: PLoS Comput Biol. 2025 Nov 13;21(11):e1013679. doi: 10.1371/journal.pcbi.1013679 (PMC12614600; doi:10.1371/journal.pcbi.1013679)
Supplement: S2 Table — (PDF) [file pcbi.1013679.s006.pdf]

Table S2: Overview of ProSPECCTs Datasets.

Dataset	Number of pockets	Dataset description
DS1	326	Structures with identical sequences but different ligands
DS1.2	45	Structures with identical sequences and similar ligands
DS2	329	Flexible NMR structures
DS3	1954	Similar structures with different physicochemical binding site
DS4	1954	Similar structures with different binding site shape properties
DS5 & 5.2	100	Similar proteins binding to identical ligands and cofactors, including phosphate binding sites (DS5.2)
DS6	35	Distant relationships between protein binding sites but identical ligands that have a similar environment
DS6.2	35	Same as in 6, additionally include cofactors
DS7	49	The recovery of known binding sites similarities within a diverse set of proteins is tested
