# Supplementary material for: OneProt: Towards multi-modal protein foundation models via latent space alignment of sequence, structure, binding sites and text encoders
Source: PLoS Comput Biol. 2025 Nov 13;21(11):e1013679. doi: 10.1371/journal.pcbi.1013679 (PMC12614600; doi:10.1371/journal.pcbi.1013679)
Supplement: S3 Table — ST. corresponds to the Structure Token modality, SG corresponds to the Structure Graph modality, ‘+’ indicates the combination of multiple modalities. (PDF) [file pcbi.1013679.s007.pdf]

Table S3: Modality alignments for selected ablations in terms of R@1 and (in parentheses) R@10. ST corresponds to Structure Token modality, SG corresponds to Structure Graph modality, '+' indicates a combination of multiple modalities.

**Algnment**	Text	Text+ST	Text+SG	Text+pocket	Text+ST +pocket	Text+SG +ST	OneProt-4 matched
seq-text	0.26 (0.68)	0.24 (0.64)	0.24 (0.63)	0.22 (0.63)	0.20 (0.60)	0.20 (0.60)	0.23 (0.62)
text-seq	0.24 (0.65)	0.22 (0.62)	0.20 (0.60)	0.20 (0.61)	0.19 (0.58)	0.18 (0.58)	0.25 (0.65)
seq-ST	-	0.45 (0.88)	-	-	0.46 (0.90)	0.48 (0.89)	-
ST-seq	-	0.43 (0.88)	-	-	0.46 (0.90)	0.45 (0.89)	-
seq-SG	-	-	0.97 (1.0)	-	-	0.84 (1.0)	0.89 (1.0)
SG-seq	-	-	0.95 (1.0)	-	-	0.84 (1.0)	0.90 (1.0)
seq-pocket	-	-	-	0.33	0.30 (0.74)	-	0.38 (0.78)
pocket-seq	-	-	-	0.30	0.28 (0.72)	-	0.35 (0.76)
SG-text	-	-	0.13 (0.47)	-	-	0.11 (0.41)	0.14 (0.49)
text-SG	-	-	0.11 (0.43)	-	-	0.10 (0.37)	0.13 (0.46)
ST-text	-	0.08 (0.35)	-	-	0.08 (0.34)	0.07 (0.31)	-
text-ST	-	0.07 (0.34)	-	-	0.07 (0.32)	0.06 (0.29)	-
pocket-text	-	-	-	0.06 (0.28)	0.05 (0.25)	-	0.07 (0.30)
text-pocket	-	-	-	0.07 (0.28)	0.05 (0.25)	-	0.07 (0.30)
pocket-SG	-	-	-	-	-	-	0.36 (0.78)
SG-pocket	-	-	-	-	-	-	0.39 (0.80)
