# Supplementary material for: OneProt: Towards multi-modal protein foundation models via latent space alignment of sequence, structure, binding sites and text encoders
Source: PLoS Comput Biol. 2025 Nov 13;21(11):e1013679. doi: 10.1371/journal.pcbi.1013679 (PMC12614600; doi:10.1371/journal.pcbi.1013679)
Supplement: S4 Table — The tasks comprise ThermoStability (regression) evaluated using Spearman correlation, HumanPPI, Metal Ion Binding, DeepLoc (binary) and DeepLoc Subcellular (multiclass classification), evaluated using accuracy (ACC) and Area Under the Receiver Operating Curve (AUC), Enzyme Commision numbers (EC), Gene Ontology (GO) terms corresponding to Molecular Function (MF), Biological Process (BP) and Cellular Component (CC) evaluated using maximum F1-score metric (Fmax) defined by Eq (6) of the main text. ST corresponds to the Structure Token modality, SG corresponds to the Structure Graph modality, ‘+’ indicates the combination of multiple modalities. (PDF) [file pcbi.1013679.s008.pdf]

Table S4: Downstream results for the ablations not included in the main text on the datasets from [17]. The tasks comprise ThermoStability (regression) evaluated using Spearman correlation, HumanPPI, Metal Ion Binding, DeepLoc Binary (binary classification) and DeepLoc Subcellular (multiclass classification), evaluated using accuracy (ACC) and Area Under the Reciever Operating Curve (AUC), Enzyme Commision numbers (EC), Gene Ontology (GO) terms corresponding to Molecular Function (MF), Biological Process (BP) and Cellular Component (CC) evaluated using maximum F1-score metric (Fmax) defined by formula (6) of the main text. ST corresponds to Structure Token modality, SG corresponds to Structure Graph modality, '+' indicates a combination of multiple modalities.

Model	Thermostability	HumanPPI	Metal Ion Binding	EC	GO			DeepLoc	
					MF	BP	CC	Subcellular	Binary
	Spearman's ρ	ACC%	ACC%	Fmax	Fmax	Fmax	Fmax	ACC%	ACC%
**Pocket**	0.601 (0.022)	75.8/84.8 (1.1/1.2)	66.1/70.8 (1.4/1.5)	0.843 (0.003)	0.604 (0.027)	0.437 (0.003)	0.473 (0.003)	62.1 (0.9)	83.6 (0.6)
**Text+Pocket**	**0.670** (0.008)	**86.1/93.7** (1.6/0.7)	72.6/83.0 (0.9/0.7)	0.872 (0.004)	**0.659** (0.003)	0.497 (0.003)	0.547 (0.006)	**81.5** (0.4)	**92.3/96.5** (0.2/0.1)
**SG**	0.616 (0.011)	77.3/84.2 (1.6/1.2)	63.9/72.3 (1.6/1.9)	0.698 (0.004)	0.467 (0.004)	0.367 (0.003)	0.447 (0.011)	62.0 (0.7)	82.8/89.5 (0.7/0.7)
**SG+Pocket**	0.616 (0.011)	81.2/87.2 (1.1/0.9)	68.2/70.8 (2.0/0.9)	0.829 (0.001)	0.589 (0.005)	0.425 (0.003)	0.473 (0.003)	63.0 (0.3)	84.0/90.4 (0.6/0.4)
**ST**	0.623 (0.018)	79.1/89.0 (1.7/0.6)	67.6/78.2 (2.5/0.4)	0.863 (0.003)	0.630 (0.005)	0.459 (0.003)	0.497 (0.011)	66.9 (0.7)	86.8/92.8 (0.4/0.2)
**ST+Pocket**	0.633 (0.016)	78.2/83.8 (2.5/1.1)	65.9/73.6 (2.0/1.2)	0.865 (0.003)	0.637 (0.004)	0.461 (0.003)	0.501 (0.009)	65.2 (0.7)	87.6/92.1 (0.5/0.3)
**ST+SG**	0.636 (0.006)	85.3/90.9 (1.4/1.8)	68.6/74.8 (0.8/2.1)	0.850 (0.003)	0.609 (0.026)	0.448 (0.002)	0.503 (0.008)	68.1 (0.7)	89.2/94.2 (0.4/0.2)
**ST+SG+Text**	0.669 (0.006)	85.0/93.1 (1.1/0.6)	**74.6/83.2** (0.3/1.2)	**0.875** (0.002)	0.654 (0.004)	0.493 (0.002)	**0.549** (0.003)	80.9 (0.4)	92.1/93.1 (0.2/0.2)
**ST+SG+Pocket**	0.642 (0.005)	77.3/85.6 (1.9/1.7)	65.5/71.8 (2.7/1.5)	0.861 (0.005)	0.627 (0.003)	0.459 (0.003)	0.498 (0.008)	66.2 (0.5)	87.9/93.1 (0.5/0.5)
**OneProt-4 matched**	0.647 (0.011)	85.7/94.5 (0.9/0.3)	74.3/81.8 (1.5/0.9)	0.867 (0.004)	0.651 (0.003)	0.491 (0.002)	0.549 (0.008)	80.8 (0.4)	91.9/96.3 (0.4/0.1)
