# Supplementary material for: OneProt: Towards multi-modal protein foundation models via latent space alignment of sequence, structure, binding sites and text encoders
Source: PLoS Comput Biol. 2025 Nov 13;21(11):e1013679. doi: 10.1371/journal.pcbi.1013679 (PMC12614600; doi:10.1371/journal.pcbi.1013679)
Supplement: S5 Table — ST corresponds to the Structure Token modality, SG corresponds to the Structure Graph modality, ‘+’ indicates the combination of multiple modalities. (PDF) [file pcbi.1013679.s009.pdf]

Table S5: Receiver Operating Characteristic Area Under the Curve (AUC) Scores for the ProSPECCTs datasets for the ablations of OneProt not included in the main text. ST corresponds to Structure Token modality, SG corresponds to Structure Graph modality, '+' indicates a combination of multiple modalities.

**Dataset**	Text	Pocket	Text+Pocket	SG	SG+Pocket	ST	ST+Pocket	ST+SG	ST+SG +Text	ST+SG +Pocket
DS1	1	1	0.9568	1	1	1	1	1	1	1
DS1.2	1	1	1	1	1	1	1	1	1	1
DS2	1	1	0.9305	1	1	1	1	1	1	1
DS3	0.7678	**0.9702**	0.8519	0.8099	0.9234	0.8648	0.9344	0.8284	0.8538	0.9051
DS4	0.7522	**0.9590**	0.8238	0.8061	0.9102	0.8408	0.9179	0.801	0.8293	0.8907
DS5 & 5.2	**0.6511**	0.5749	0.6436	0.5434	0.5535	0.5608	0.5741	0.5485	0.6375	0.5675
DS6	0.5605	0.5828	0.5806	0.46	0.5684	0.6142	**0.6187**	0.5935	0.6108	0.6139
DS6.2	0.5602	0.5828	0.5805	0.4598	0.5683	0.6141	**0.6187**	0.5933	0.6107	0.6139
DS7	0.8469	0.8193	**0.8487**	0.7552	0.7967	0.7723	0.8011	0.7446	0.8398	0.7928
