# Supplementary material for: OneProt: Towards multi-modal protein foundation models via latent space alignment of sequence, structure, binding sites and text encoders
Source: PLoS Comput Biol. 2025 Nov 13;21(11):e1013679. doi: 10.1371/journal.pcbi.1013679 (PMC12614600; doi:10.1371/journal.pcbi.1013679)
Supplement: S6 Table — For the last two columns, concatenated embeddings are used for prediction. (PDF) [file pcbi.1013679.s010.pdf]

Table S6: Receiver Operating Characteristic Area Under the Curve (AUC) Scores for the ProSPECCTs datasets using alternative to sequence embeddings from OneProt-5. For the last two columns concatenated embeddings are used for prediction.

**Dataset**	Structure Only	Pocket Only	Sequence & Structure	Sequence, Structure & Pocket
DS1	1.000	1.000	1.000	1.000
DS1.2	1.000	1.000	1.000	1.000
DS2	1.000	0.998	0.998	1.000
DS3	**0.531**	0.495	0.495	0.505
DS4	**0.692**	0.620	0.620	0.673
DS5	0.603	0.598	**0.599**	0.613
DS6	0.570	0.566	0.567	0.565
DS6.2	0.573	0.577	**0.578**	0.571
DS7	0.795	**0.856**	**0.856**	0.839
