# Supplementary material for: OneProt: Towards multi-modal protein foundation models via latent space alignment of sequence, structure, binding sites and text encoders
Source: PLoS Comput Biol. 2025 Nov 13;21(11):e1013679. doi: 10.1371/journal.pcbi.1013679 (PMC12614600; doi:10.1371/journal.pcbi.1013679)
Supplement: S7 Table — Q1 and Q3 correspond to the first and third quartiles of the data, respectively. Column outliers correspond to the count of values below Q1-1.5×IQR. (PDF) [file pcbi.1013679.s011.pdf]

Table S7: Numerical values corresponding to boxplots in Figs 4 and S5. Q1 and Q3 correspond to the first and third quartiles of the data respectively. Column outliers correspond to the count of values below Q1-1.5×IQR. ST corresponds to Structure Token modality, SG corresponds to Structure Graph modality, '+' indicates a combination of multiple modalities.

Model	Q1	Median	Q3	IQR	Outliers	Total Points
TopEC	0.50	0.75	0.97	0.47	0	826
CLEAN	0.57	0.95	1.00	0.43	0	826
ESM2	0.67	0.94	1.00	0.33	50	826
ESM-IF	0.62	0.92	1.00	0.38	55	826
OpenFold	0.69	0.95	1.00	0.31	54	826
ProTrek-35M	0.61	0.89	0.99	0.38	52	826
ProTrek-650M	0.75	0.95	1.00	0.25	58	826
ST+SG+Pocket+Text (OneProt-5)	0.71	0.97	1.00	0.29	51	826
SG+Pocket+Text (OneProt-4)	0.70	0.96	1.00	0.30	53	826
Text Only	0.70	0.94	1.00	0.30	60	826
Pocket+Text	0.70	0.96	1.00	0.30	58	826
SG+Text	0.68	0.95	1.00	0.32	53	826
ST+Text	0.70	0.96	1.00	0.30	62	826
ST+SG+Text	0.72	0.95	1.00	0.28	46	826
ST+SG+Pocket	0.70	0.94	1.00	0.30	51	826
ST+Pocket+Text	0.73	0.96	1.00	0.27	55	826
ST+Pocket	0.71	0.96	1.00	0.29	52	826
ST Only	0.68	0.93	1.00	0.32	68	826
ST+SG	0.62	0.93	1.00	0.38	67	826
Pocket Only	0.65	0.92	1.00	0.35	55	826
SG+Pocket	0.61	0.91	1.00	0.39	62	826
