# Supplementary material for: OneProt: Towards multi-modal protein foundation models via latent space alignment of sequence, structure, binding sites and text encoders
Source: PLoS Comput Biol. 2025 Nov 13;21(11):e1013679. doi: 10.1371/journal.pcbi.1013679 (PMC12614600; doi:10.1371/journal.pcbi.1013679)
Supplement: S8 Table — (PDF) [file pcbi.1013679.s012.pdf]

Table S8: Table of ranges (Min, Max), 0.25 (Q1), 0.5 (Median), 0.75 (Q3), Inter Quantile Range (IQR = Q3 - Q1) for metrics of different models on DeepLoc2, DeepLoc10 (accuracy), EC (Fmax) tasks. Task and modality names as in S4 Table.

**DeepLoc2 Accuracy**	**Min**	**Q1**	**Median**	**Q3**	**Max**	**IQR**
OnepProt-5	0.92	0.923	0.924	0.925	0.925	0.002
Text Only	0.926	0.928	0.928	0.931	0.933	0.003
Pocket Only	0.826	0.831	0.837	0.840	0.842	0.009
Pocket+Text	0.92	0.922	0.923	0.925	0.926	0.003
SG only	0.813	0.825	0.830	0.834	0.835	0.009
SG+Text	0.923	0.927	0.929	0.932	0.934	0.005
SG+Pocket	0.833	0.835	0.837	0.846	0.848	0.011
OneProt-4	0.916	0.920	0.921	0.924	0.928	0.004
ST only	0.864	0.865	0.867	0.868	0.876	0.003
ST+Text	0.923	0.925	0.929	0.929	0.931	0.004
ST+Pocket	0.868	0.873	0.876	0.88	0.881	0.007
ST+Pocket+Text	0.908	0.912	0.913	0.916	0.916	0.004
ST+SG	0.885	0.888	0.891	0.895	0.897	0.007
ST+SG+Text	0.917	0.920	0.921	0.921	0.924	0.001
ST+SG+Pocket	0.872	0.874	0.880	0.883	0.885	0.009
ProTrek-35M	0.924	0.929	0.933	0.933	0.935	0.004
ProTrek-650M	0.95	0.951	0.953	0.954	0.955	0.003
ESM-2	0.906	0.911	0.915	0.916	0.918	0.005
SaProt	0.911	0.907	0.910	0.913	0.913	0.006
ESM-3	0.905	0.906	0.906	0.908	0.91	0.002
ESM-IF	0.838	0.844	0.848	0.848	0.854	0.004
OpenFold	0.912	0.914	0.918	0.92	0.921	0.006
OneProt-4 matched	0.913	0.914	0.918	0.922	0.926	0.008
**DeepLoc10 Accuracy**	**Min**	**Q1**	**Median**	**Q3**	**Max**	**IQR**
OnepProt-5	0.799	0.801	0.802	0.805	0.806	0.004
Text Only	0.823	0.828	0.829	0.834	0.834	0.006
Pocket Only	0.604	0.617	0.6245	0.628	0.629	0.011
Pocket+Text	0.809	0.812	0.816	0.819	0.821	0.007
SG only	0.608	0.615	0.620	0.627	0.628	0.012
SG+Text	0.817	0.820	0.823	0.824	0.828	0.004
SG+Pocket	0.623	0.629	0.631	0.631	0.632	0.003
OneProt-4	0.812	0.813	0.819	0.820	0.821	0.007
ST only	0.658	0.665	0.670	0.671	0.680	0.006
ST+Text	0.809	0.814	0.818	0.818	0.819	0.004
ST+Pocket	0.64	0.649	0.653	0.657	0.659	0.008
ST+Pocket+Text	0.798	0.802	0.805	0.808	0.812	0.006
ST+SG	0.67	0.675	0.683	0.686	0.69	0.011
ST+SG+Text	0.803	0.807	0.810	0.813	0.814	0.006
ST+SG+Pocket	0.656	0.658	0.662	0.664	0.669	0.006
ProTrek-35M	0.834	0.835	0.836	0.839	0.844	0.004
ProTrek-650M	0.902	0.908	0.911	0.911	0.913	0.003
ESM-2	0.803	0.807	0.811	0.813	0.813	0.006
SaProt	0.783	0.788	0.791	0.795	0.795	0.007
ESM-3	0.756	0.759	0.763	0.765	0.77	0.006
ESM-IF	0.609	0.609	0.613	0.618	0.627	0.009
OpenFold	0.791	0.798	0.800	0.802	0.805	0.004
OneProt-4 matched	0.800	0.807	0.809	0.811	0.812	0.004
**EC Fmax**	**Min**	**Q1**	**Median**	**Q3**	**Max**	**IQR**
OnepProt-5	0.869	0.870	0.872	0.879	0.884	0.009
Text Only	0.872	0.874	0.875	0.876	0.882	0.002
Pocket Only	0.839	0.841	0.843	0.844	0.849	0.003
Pocket+Text	0.867	0.868	0.873	0.875	0.876	0.007
SG only	0.691	0.696	0.700	0.701	0.703	0.005
SG+Text	0.86	0.864	0.868	0.869	0.871	0.005
SG+Pocket	0.826	0.829	0.829	0.83	0.83	0.001
OneProt-4	0.867	0.8685	0.871	0.873	0.876	0.0045
ST only	0.859	0.861	0.862	0.865	0.868	0.004
ST+Text	0.871	0.876	0.878	0.878	0.882	0.002
ST+Pocket	0.861	0.863	0.866	0.867	0.868	0.004
ST+Pocket+Text	0.871	0.872	0.8765	0.879	0.881	0.007
ST+SG	0.845	0.847	0.851	0.852	0.853	0.005
ST+SG+Text	0.873	0.874	0.875	0.878	0.878	0.004
ST+SG+Pocket	0.855	0.858	0.861	0.865	0.869	0.007
ProTrek-35M	0.841	0.845	0.846	0.847	0.850	0.002
ProTrek-650M	0.867	0.873	0.878	0.879	0.880	0.006
ESM-2	0.874	0.876	0.878	0.879	0.885	0.003
SaProt	0.856	0.862	0.863	0.866	0.867	0.004
ESM-3	0.865	0.870	0.871	0.873	0.877	0.003
ESM-IF	0.886	0.895	0.897	0.899	0.905	0.004
OpenFold	0.882	0.885	0.889	0.889	0.892	0.004
OneProt-4 matched	0.862	0.864	0.869	0.870	0.872	0.006
