# Supplementary material for: OneProt: Towards multi-modal protein foundation models via latent space alignment of sequence, structure, binding sites and text encoders
Source: PLoS Comput Biol. 2025 Nov 13;21(11):e1013679. doi: 10.1371/journal.pcbi.1013679 (PMC12614600; doi:10.1371/journal.pcbi.1013679)
Supplement: S9 Table — (PDF) [file pcbi.1013679.s013.pdf]

Table S9: Table of ranges (Min, Max), 0.25 (Q1), 0.5 (Median), 0.75 (Q3), Inter Quantile Range (IQR = Q3 - Q1) for metrics of different models on GO-BP, GO-CC, GO-MF (Fmax) tasks.Task and modality names as in S4 Table.

**GO-BP Fmax**	**Min**	**Q1**	**Median**	**Q3**	**Max**	**IQR**
OnepProt-5	0.486	0.491	0.492	0.493	0.496	0.002
Text Only	0.499	0.502	0.503	0.504	0.505	0.002
Pocket Only	0.433	0.435	0.437	0.439	0.441	0.004
Pocket+Text	0.494	0.494	0.497	0.499	0.500	0.005
SG only	0.362	0.364	0.367	0.369	0.370	0.005
SG+Text	0.492	0.492	0.495	0.499	0.499	0.007
SG+Pocket	0.419	0.425	0.426	0.426	0.428	0.001
OneProt-4	0.491	0.493	0.494	0.496	0.500	0.003
ST only	0.456	0.456	0.458	0.460	0.466	0.004
ST+Text	0.491	0.495	0.496	0.498	0.498	0.003
ST+Pocket	0.458	0.458	0.460	0.462	0.466	0.004
ST+Pocket+Text	0.493	0.494	0.496	0.498	0.503	0.004
ST+SG	0.446	0.447	0.449	0.449	0.451	0.002
ST+SG+Text	0.491	0.491	0.493	0.494	0.495	0.003
ST+SG+Pocket	0.457	0.457	0.458	0.461	0.466	0.004
ProTrek-35M	0.504	0.513	0.515	0.518	0.519	0.005
ProTrek-650M	0.53	0.537	0.538	0.541	0.543	0.004
ESM-2	0.474	0.476	0.479	0.481	0.483	0.005
SaProt	0.469	0.472	0.473	0.474	0.477	0.002
ESM-3	0.478	0.48	0.481	0.483	0.488	0.003
ESM-IF	0.432	0.432	0.438	0.439	0.44	0.007
OpenFold	0.487	0.491	0.492	0.492	0.493	0.001
OneProt-4 matched	0.488	0.488	0.491	0.494	0.494	0.006
**GO-CC Fmax**	**Min**	**Q1**	**Median**	**Q3**	**Max**	**IQR**
OnepProt-5	0.551	0.552	0.556	0.56	0.562	0.008
Text Only	0.553	0.556	0.563	0.564	0.567	0.008
Pocket Only	0.471	0.471	0.472	0.475	0.478	0.004
Pocket+Text	0.537	0.545	0.545	0.551	0.554	0.006
SG only	0.428	0.442	0.448	0.454	0.462	0.0115
SG+Text	0.541	0.543	0.5435	0.545	0.552	0.002
SG+Pocket	0.469	0.471	0.4735	0.476	0.477	0.005
OneProt-4	0.544	0.552	0.555	0.558	0.565	0.006
ST only	0.482	0.489	0.495	0.504	0.517	0.015
ST+Text	0.54	0.545	0.548	0.554	0.559	0.009
ST+Pocket	0.485	0.498	0.499	0.507	0.518	0.009
ST+Pocket+Text	0.541	0.541	0.546	0.549	0.55	0.008
ST+SG	0.495	0.496	0.502	0.512	0.513	0.016
ST+SG+Text	0.545	0.547	0.548	0.549	0.554	0.002
ST+SG+Pocket	0.491	0.493	0.494	0.504	0.512	0.011
ProTrek-35M	0.568	0.58	0.583	0.589	0.594	0.009
ProTrek-650M	0.611	0.611	0.618	0.621	0.623	0.01
ESM-2	0.543	0.548	0.551	0.552	0.556	0.004
SaProt	0.54	0.548	0.549	0.550	0.554	0.002
ESM-3	0.508	0.529	0.531	0.536	0.541	0.007
ESM-IF	0.479	0.485	0.489	0.491	0.496	0.006
OpenFold	0.541	0.546	0.548	0.551	0.553	0.005
OneProt-4 matched	0.534	0.544	0.548	0.557	0.559	0.013
**GO-MF Fmax**	**Min**	**Q1**	**Median**	**Q3**	**Max**	**IQR**
OnepProt-5	0.653	0.654	0.656	0.657	0.659	0.003
Text Only	0.652	0.653	0.659	0.660	0.660	0.007
Pocket Only	0.600	0.604	0.605	0.608	0.608	0.004
Pocket+Text	0.652	0.659	0.660	0.661	0.662	0.002
SG only	0.463	0.464	0.467	0.472	0.474	0.008
SG+Text	0.647	0.647	0.651	0.653	0.659	0.006
SG+Pocket	0.583	0.584	0.590	0.593	0.596	0.009
OneProt-4	0.654	0.655	0.656	0.656	0.656	0.001
ST only	0.622	0.628	0.632	0.632	0.637	0.004
ST+Text	0.657	0.659	0.661	0.663	0.665	0.004
ST+Pocket	0.632	0.636	0.637	0.638	0.643	0.002
ST+Pocket+Text	0.648	0.656	0.657	0.657	0.664	0.001
ST+SG	0.607	0.607	0.608	0.612	0.613	0.005
ST+SG+Text	0.647	0.652	0.655	0.657	0.658	0.005
ST+SG+Pocket	0.623	0.624	0.627	0.628	0.631	0.004
ProTrek-35M	0.648	0.649	0.651	0.653	0.653	0.004
ProTrek-650M	0.669	0.669	0.676	0.678	0.684	0.009
ESM-2	0.64	0.643	0.645	0.647	0.649	0.004
SaProt	0.619	0.620	0.624	0.628	0.635	0.008
ESM-3	0.63	0.640	0.642	0.644	0.648	0.004
ESM-IF	0.606	0.608	0.610	0.613	0.619	0.005
OpenFold	0.649	0.652	0.655	0.657	0.664	0.005
OneProt-4 matched	0.648	0.648	0.652	0.653	0.656	0.005
