# Supplementary material for: OneProt: Towards multi-modal protein foundation models via latent space alignment of sequence, structure, binding sites and text encoders
Source: PLoS Comput Biol. 2025 Nov 13;21(11):e1013679. doi: 10.1371/journal.pcbi.1013679 (PMC12614600; doi:10.1371/journal.pcbi.1013679)
Supplement: S10 Table — (PDF) [file pcbi.1013679.s014.pdf]

Table S10: Table of ranges (Min, Max), 0.25 (Q1), 0.5 (Median), 0.75 (Q3), Inter Quantile Range (IQR = Q3 - Q1) for metrics of different models on HumanPPI, Metal Ion Binding (accuracy), ThermoStability (Spearman's ρ) tasks. Task and modality names as in S4 Table.

**HumanPPI**						
	**Min**	**Q1**	**Median**	**Q3**	**Max**	**IQR**
OnepProt-5	0.811	0.856	0.867	0.872	0.883	0.016
Text Only	0.856	0.861	0.875	0.883	0.900	0.022
Pocket Only	0.744	0.75	0.759	0.767	0.772	0.017
Pocket+Text	0.833	0.856	0.861	0.867	0.894	0.011
SG only	0.756	0.761	0.767	0.794	0.794	0.033
SG+Text	0.833	0.856	0.872	0.883	0.889	0.027
SG+Pocket	0.800	0.800	0.811	0.822	0.828	0.022
OneProt-4	0.85	0.889	0.889	0.900	0.906	0.011
ST only	0.767	0.783	0.794	0.806	0.817	0.023
ST+Text	0.817	0.838	0.844	0.856	0.861	0.018
ST+Pocket	0.75	0.771	0.772	0.794	0.833	0.023
ST+Pocket+Text	0.833	0.844	0.861	0.867	0.883	0.023
ST+SG	0.828	0.844	0.856	0.861	0.872	0.017
ST+SG+Text	0.833	0.844	0.85	0.856	0.872	0.012
ST+SG+Pocket	0.756	0.761	0.767	0.783	0.806	0.022
ProTrek-35M	0.839	0.856	0.859	0.878	0.894	0.022
ProTrek-650M	0.878	0.894	0.906	0.911	0.928	0.017
ESM-2	0.844	0.855	0.861	0.868	0.872	0.013
SaProt	0.850	0.861	0.870	0.883	0.900	0.022
ESM-3	0.811	0.825	0.834	0.847	0.861	0.022
ESM-IF	0.756	0.782	0.794	0.794	0.806	0.012
OpenFold	0.806	0.817	0.85	0.861	0.867	0.044
OneProt-4 matched	0.839	0.855	0.859	0.863	0.867	0.008
**MetalIonBinding**						
	**Min**	**Q1**	**Median**	**Q3**	**Max**	**IQR**
OnepProt-5	0.732	0.753	0.767	0.770	0.782	0.017
Text Only	0.709	0.719	0.754	0.759	0.762	0.040
Pocket Only	0.642	0.650	0.66	0.675	0.677	0.025
Pocket+Text	0.72	0.722	0.723	0.725	0.744	0.003
SG only	0.615	0.630	0.640	0.645	0.662	0.015
SG+Text	0.746	0.752	0.763	0.764	0.768	0.012
SG+Pocket	0.656	0.674	0.682	0.683	0.716	0.009
OneProt-4	0.765	0.770	0.774	0.776	0.780	0.006
ST only	0.653	0.657	0.668	0.692	0.719	0.035
ST+Text	0.731	0.741	0.747	0.756	0.762	0.015
ST+Pocket	0.632	0.643	0.660	0.675	0.687	0.032
ST+Pocket+Text	0.729	0.740	0.744	0.755	0.758	0.015
ST+SG	0.675	0.678	0.683	0.695	0.696	0.017
ST+SG+Text	0.743	0.744	0.747	0.748	0.75	0.004
ST+SG+Pocket	0.626	0.638	0.642	0.678	0.701	0.040
ProTrek-35M	0.746	0.749	0.765	0.770	0.771	0.021
ProTrek-650M	0.692	0.749	0.765	0.779	0.783	0.030
ESM-2	0.659	0.663	0.673	0.683	0.693	0.020
SaProt	0.686	0.708	0.716	0.720	0.732	0.012
ESM-3	0.696	0.707	0.737	0.741	0.744	0.034
ESM-IF	0.672	0.680	0.689	0.696	0.711	0.016
OpenFold	0.708	0.713	0.717	0.723	0.731	0.010
OneProt-4 matched	0.713	0.743	0.744	0.755	0.761	0.012
**ThermoStability**						
	**Min**	**Q1**	**Median**	**Q3**	**Max**	**IQR**
OnepProt-5	0.657	0.668	0.676	0.681	0.682	0.013
Text Only	0.65	0.651	0.656	0.66	0.663	0.009
Pocket Only	0.572	0.573	0.601	0.619	0.628	0.046
Pocket+Text	0.658	0.667	0.673	0.675	0.679	0.008
SG only	0.612	0.614	0.616	0.618	0.623	0.004
SG+Text	0.640	0.657	0.666	0.674	0.681	0.007
SG+Pocket	0.64	0.657	0.666	0.674	0.681	0.017
OneProt-4	0.656	0.668	0.670	0.672	0.673	0.004
ST only	0.589	0.619	0.6285	0.636	0.639	0.017
ST+Text	0.638	0.666	0.6715	0.674	0.677	0.008
ST+Pocket	0.604	0.630	0.636	0.644	0.648	0.014
ST+Pocket+Text	0.665	0.666	0.669	0.671	0.683	0.005
ST+SG	0.628	0.630	0.635	0.644	0.648	0.014
ST+SG+Text	0.66	0.665	0.672	0.674	0.675	0.009
ST+SG+Pocket	0.632	0.638	0.644	0.646	0.646	0.008
ProTrek-35M	0.623	0.629	0.637	0.647	0.653	0.018
ProTrek-650M	0.628	0.643	0.649	0.65	0.655	0.007
ESM-2	0.690	0.692	0.696	0.700	0.703	0.008
SaProt	0.693	0.699	0.701	0.705	0.71	0.006
ESM-3	0.669	0.686	0.688	0.702	0.712	0.016
ESM-IF	0.637	0.638	0.645	0.650	0.653	0.012
OpenFold	0.567	0.571	0.580	0.590	0.601	0.019
OneProt-4 matched	0.635	0.642	0.645	0.650	0.668	0.008
