# Supplementary material for: OneProt: Towards multi-modal protein foundation models via latent space alignment of sequence, structure, binding sites and text encoders
Source: PLoS Comput Biol. 2025 Nov 13;21(11):e1013679. doi: 10.1371/journal.pcbi.1013679 (PMC12614600; doi:10.1371/journal.pcbi.1013679)
Supplement: S11 Table — (PDF) [file pcbi.1013679.s015.pdf]

Table S11: Table of ranges (Min, Max), 0.25 (Q1), 0.5 (Median), 0.75 (Q3), Inter Quantile Range (IQR = Q3 - Q1) for AUC of different models on binary classification tasks: DeepLoc2, HumanPPI, Metal IonBinding) tasks. Task and modality names as in S4 Table.

**DeepLoc2 AUC**	**Min**	**Q1**	**Median**	**Q3**	**Max**	**IQR**
OnepProt-5	0.960	0.962	0.964	0.964	0.967	0.002
Text Only	0.971	0.972	0.973	0.974	0.975	0.002
Pocket Only	0.894	0.898	0.900	0.902	0.903	0.005
Pocket+Text	0.961	0.963	0.964	0.966	0.968	0.003
SG only	0.889	0.893	0.896	0.900	0.903	0.008
SG+Text	0.964	0.967	0.970	0.972	0.973	0.005
SG+Pocket	0.898	0.901	0.906	0.907	0.909	0.006
OneProt-4	0.960	0.964	0.965	0.967	0.969	0.003
ST only	0.924	0.926	0.929	0.929	0.930	0.003
ST+Text	0.967	0.968	0.969	0.969	0.970	0.001
ST+Pocket	0.916	0.920	0.922	0.922	0.928	0.002
ST+Pocket+Text	0.960	0.961	0.963	0.965	0.966	0.004
ST+SG	0.940	0.941	0.943	0.944	0.944	0.003
ST+SG+Text	0.964	0.967	0.970	0.971	0.971	0.004
ST+SG+Pocket	0.928	0.930	0.930	0.932	0.934	0.002
ProTrek-35M	0.975	0.977	0.978	0.979	0.981	0.002
ProTrek-650M	0.986	0.988	0.988	0.989	0.990	0.001
ESM-2	0.959	0.963	0.964	0.965	0.968	0.002
SaProt	0.913	0.958	0.959	0.961	0.963	0.003
ESM-3	0.955	0.956	0.957	0.958	0.960	0.002
ESM-IF	0.902	0.904	0.904	0.906	0.909	0.002
OpenFold	0.962	0.965	0.966	0.968	0.968	0.003
**HumanPPI AUC**	**Min**	**Q1**	**Median**	**Q3**	**Max**	**IQR**
OnepProt-5	0.924	0.929	0.935	0.940	0.944	0.011
Text Only	0.938	0.941	0.942	0.942	0.953	0.001
Pocket Only	0.833	0.838	0.849	0.858	0.860	0.020
Pocket+Text	0.929	0.932	0.937	0.940	0.948	0.008
SG only	0.821	0.837	0.844	0.849	0.856	0.012
SG+Text	0.957	0.961	0.963	0.965	0.970	0.004
SG+Pocket	0.860	0.866	0.875	0.879	0.882	0.013
OneProt-4	0.949	0.952	0.954	0.956	0.956	0.004
ST only	0.882	0.885	0.889	0.892	0.902	0.007
ST+Text	0.916	0.920	0.925	0.934	0.943	0.014
ST+Pocket	0.824	0.829	0.841	0.846	0.849	0.017
ST+Pocket+Text	0.930	0.938	0.939	0.940	0.949	0.002
ST+SG	0.889	0.896	0.902	0.927	0.930	0.031
ST+SG+Text	0.920	0.928	0.932	0.933	0.942	0.004
ST+SG+Pocket	0.834	0.841	0.862	0.867	0.873	0.026
ProTrek-35M	0.935	0.940	0.944	0.950	0.954	0.011
ProTrek-650M	0.962	0.968	0.972	0.973	0.977	0.005
ESM-2	0.934	0.938	0.941	0.947	0.952	0.010
SaProt	0.863	0.936	0.939	0.942	0.944	0.005
ESM-3	0.912	0.914	0.920	0.924	0.943	0.009
ESM-IF	0.854	0.856	0.860	0.863	0.871	0.007
OpenFold	0.910	0.912	0.925	0.929	0.933	0.017
**Metal Ion Binding AUC AUC**	**Min**	**Q1**	**Median**	**Q3**	**Max**	**IQR**
OnepProt-5	0.919	0.926	0.930	0.934	0.940	0.008
Text Only	0.938	0.941	0.942	0.951	0.956	0.010
Pocket Only	0.825	0.838	0.845	0.852	0.859	0.014
Pocket+Text	0.929	0.932	0.937	0.940	0.948	0.008
SG only	0.809	0.832	0.846	0.855	0.858	0.023
SG+Text	0.951	0.961	0.963	0.965	0.970	0.004
SG+Pocket	0.860	0.866	0.875	0.879	0.882	0.013
OneProt-4	0.938	0.940	0.942	0.944	0.952	0.005
ST only	0.874	0.882	0.885	0.892	0.902	0.010
ST+Text	0.916	0.920	0.922	0.928	0.965	0.007
ST+Pocket	0.813	0.826	0.844	0.865	0.876	0.040
ST+Pocket+Text	0.933	0.938	0.939	0.943	0.949	0.005
ST+SG	0.889	0.895	0.913	0.925	0.930	0.030
ST+SG+Text	0.928	0.933	0.934	0.940	0.945	0.007
ST+SG+Pocket	0.836	0.859	0.867	0.872	0.881	0.012
ProTrek-35M	0.926	0.936	0.938	0.945	0.954	0.009
ProTrek-650M	0.963	0.970	0.973	0.974	0.977	0.003
ESM-2	0.937	0.939	0.947	0.948	0.952	0.009
SaProt	0.863	0.936	0.939	0.940	0.941	0.004
ESM-3	0.901	0.914	0.920	0.925	0.943	0.011
ESM-IF	0.844	0.852	0.855	0.858	0.866	0.006
OpenFold	0.914	0.919	0.926	0.929	0.933	0.010
