# Supplementary material for: OneProt: Towards multi-modal protein foundation models via latent space alignment of sequence, structure, binding sites and text encoders
Source: PLoS Comput Biol. 2025 Nov 13;21(11):e1013679. doi: 10.1371/journal.pcbi.1013679 (PMC12614600; doi:10.1371/journal.pcbi.1013679)
Supplement: S2 Fig — The alternative hypothesis of OneProt models (vertical axis) performing differently from baseline models (horizontal axis) according to metric values from Tables 3 and 4, where for binary classification accuracy was compared. Striped pattern stands for the values p≥0.05, when the null hypothesis of OneProt being the same as the baseline was not rejected. (PDF) [file pcbi.1013679.s017.pdf]

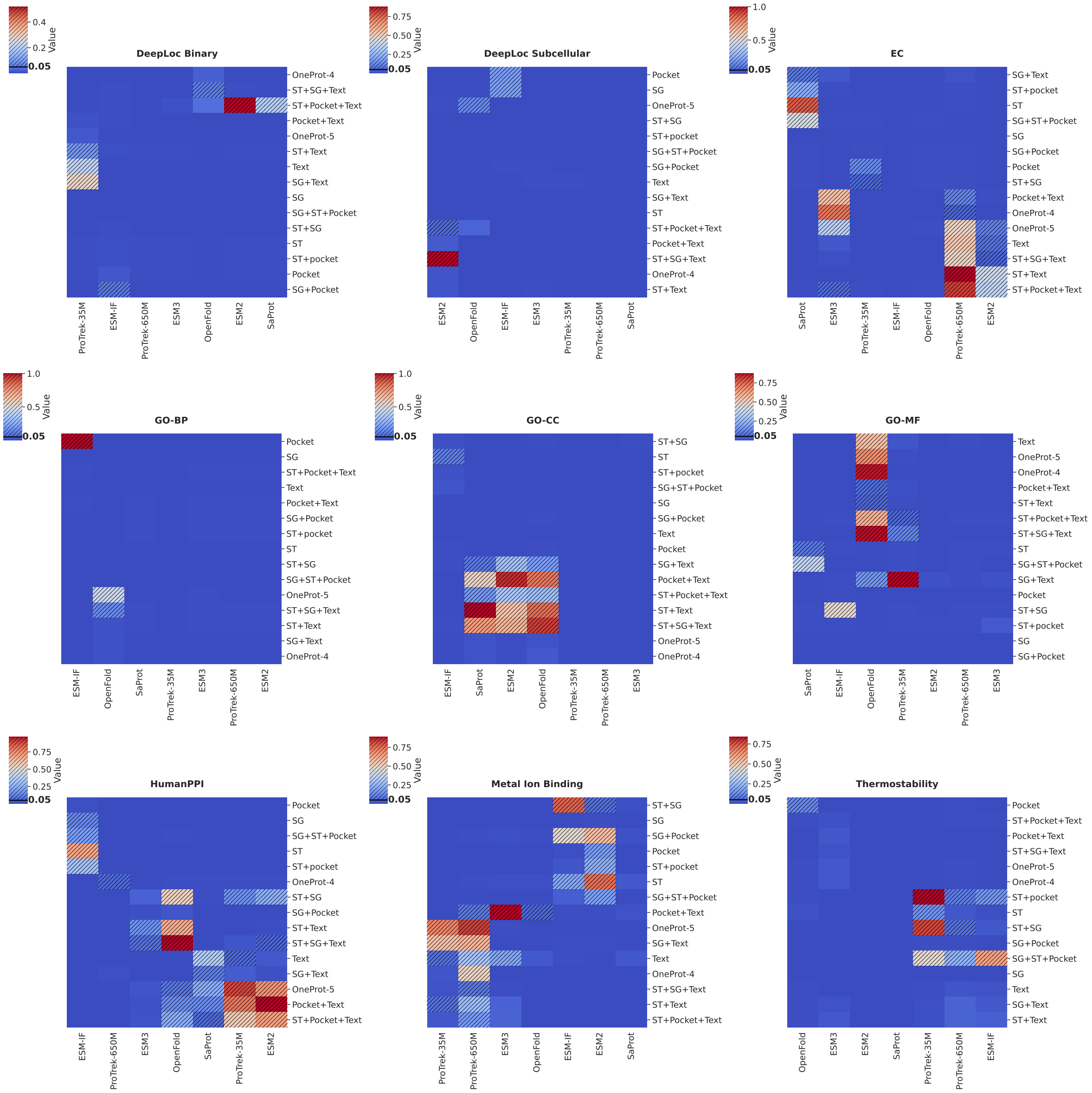

Figure S2: **Heatmaps of p-values according to two-sided Wilcoxon rank-sum test.** The alternative hypothesis of OneProt models (vertical axis) performing differently from baseline models (horizontal axis) according to metric values from Tables 3 and 4 of the main text, where for binary classification accuracy was compared. Striped pattern stands for the values $p \geq 0.05$, when the null hypothesis of OneProt being the same as the baseline was not rejected.
