# Supplementary material for: OneProt: Towards multi-modal protein foundation models via latent space alignment of sequence, structure, binding sites and text encoders
Source: PLoS Comput Biol. 2025 Nov 13;21(11):e1013679. doi: 10.1371/journal.pcbi.1013679 (PMC12614600; doi:10.1371/journal.pcbi.1013679)
Supplement: S3 Fig — One-sided Wilcoxon rank-sum test with the alternative hypothesis of OneProt (vertical axis) outperforming baseline models (horizontal axis), striped pattern corresponding to values p < 0.05 (upper panel). Two-sided Wilcoxon rank-sum test with the alternative hypothesis of OneProt performing differently than baseline models, striped pattern corresponding to values p≥0.05 (middle panel). One-sided Wilcoxon rank-sum test for OneProt ablations with the alternative hypothesis of the models on the vertical axis outperforming the models on the horizontal axis (bottom panel). Striped pattern stands for the values p < 0.05. (PDF) [file pcbi.1013679.s018.pdf]

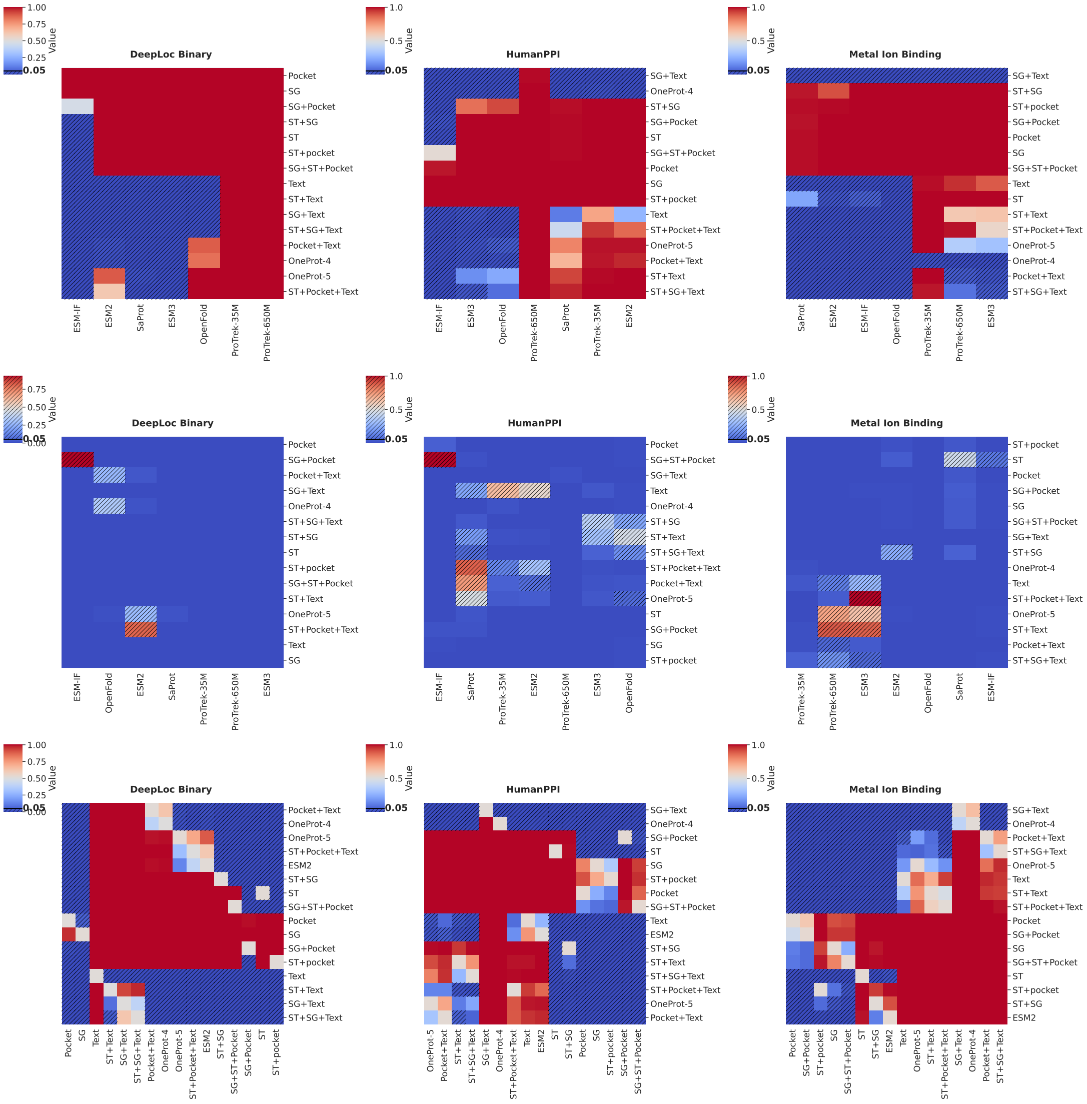

Figure S3: **Heatmaps of p-values for Area Under Receiver Operating Characteristic curve metrics.** One-sided Wilcoxon rank-sum test with the alternative hypothesis of OneProt (vertical axis) outperforming baseline models (horizontal axis), striped pattern corresponding to values $p < 0.05$ (upper panel). Two-sided Wilcoxon rank-sum test with the alternative hypothesis of OneProt performing differently than baseline models, striped pattern corresponding to values $p \geq 0.05$ (middle panel). One-sided Wilcoxon rank-sum test for OneProt ablations with the alternative hypothesis of the models on the vertical axis outperforming the models on the horizontal axis (bottom panel). Striped pattern stands for the values $p < 0.05$.
