# Supplementary material for: OneProt: Towards multi-modal protein foundation models via latent space alignment of sequence, structure, binding sites and text encoders
Source: PLoS Comput Biol. 2025 Nov 13;21(11):e1013679. doi: 10.1371/journal.pcbi.1013679 (PMC12614600; doi:10.1371/journal.pcbi.1013679)
Supplement: S6 Fig — The plot shows the similarity of a given protein to three groups: the 50 most evolutionarily similar proteins, the 50 most evolutionarily divergent sequences, and 1000 unrelated sequences. ST corresponds to the Structure Token modality, SG corresponds to the Structure Graph modality, ‘+’ indicates the combination of multiple modalities. (PDF) [file pcbi.1013679.s021.pdf]

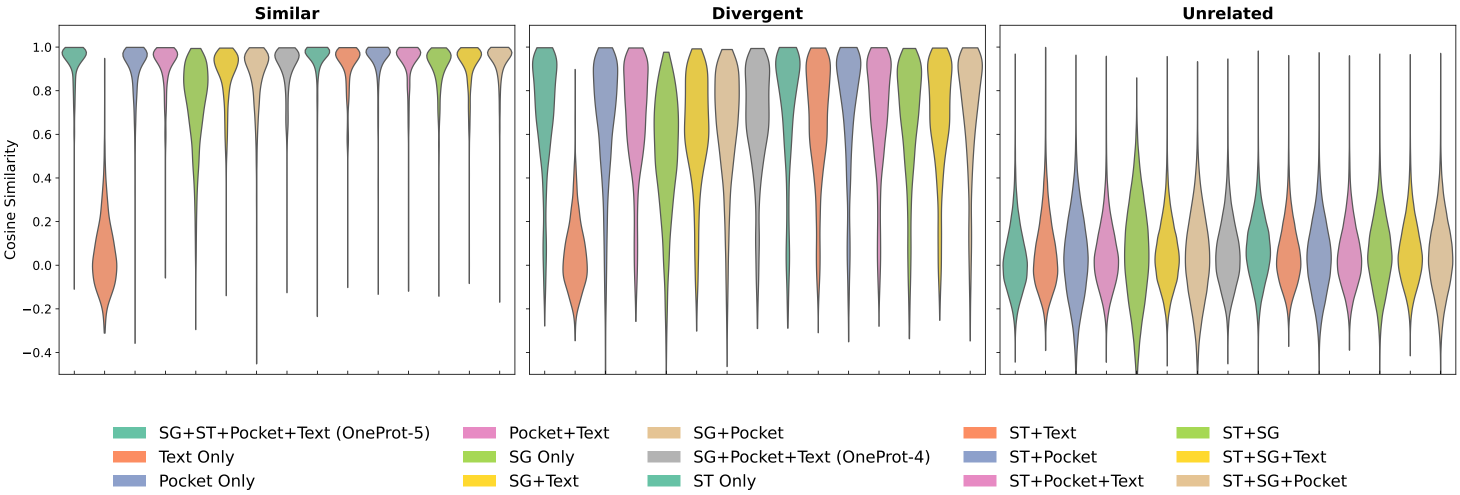

Figure S6: Cosine Similarity distributions for OneProt ablations. The plot shows the similarity of a given protein to three groups: the 50 most evolutionarily similar proteins, the 50 most evolutionarily divergent sequences, and 1000 unrelated sequences.ST corresponds to Structure Token modality, SG corresponds to Structure Graph modality, '+' indicates a combination of multiple modalities.
