# Supplementary material for: OneProt: Towards multi-modal protein foundation models via latent space alignment of sequence, structure, binding sites and text encoders
Source: PLoS Comput Biol. 2025 Nov 13;21(11):e1013679. doi: 10.1371/journal.pcbi.1013679 (PMC12614600; doi:10.1371/journal.pcbi.1013679)
Supplement: S4 Fig — The alternative hypothesis of the models on the vertical axis outperforming the models on the horizontal according to metric values from Tables 3 and 4, where for binary classification accuracy was compared. Striped pattern stands for the values p < 0.05, when the null hypothesis of model on the vertical axis being non-superior was rejected. (PDF) [file pcbi.1013679.s019.pdf]

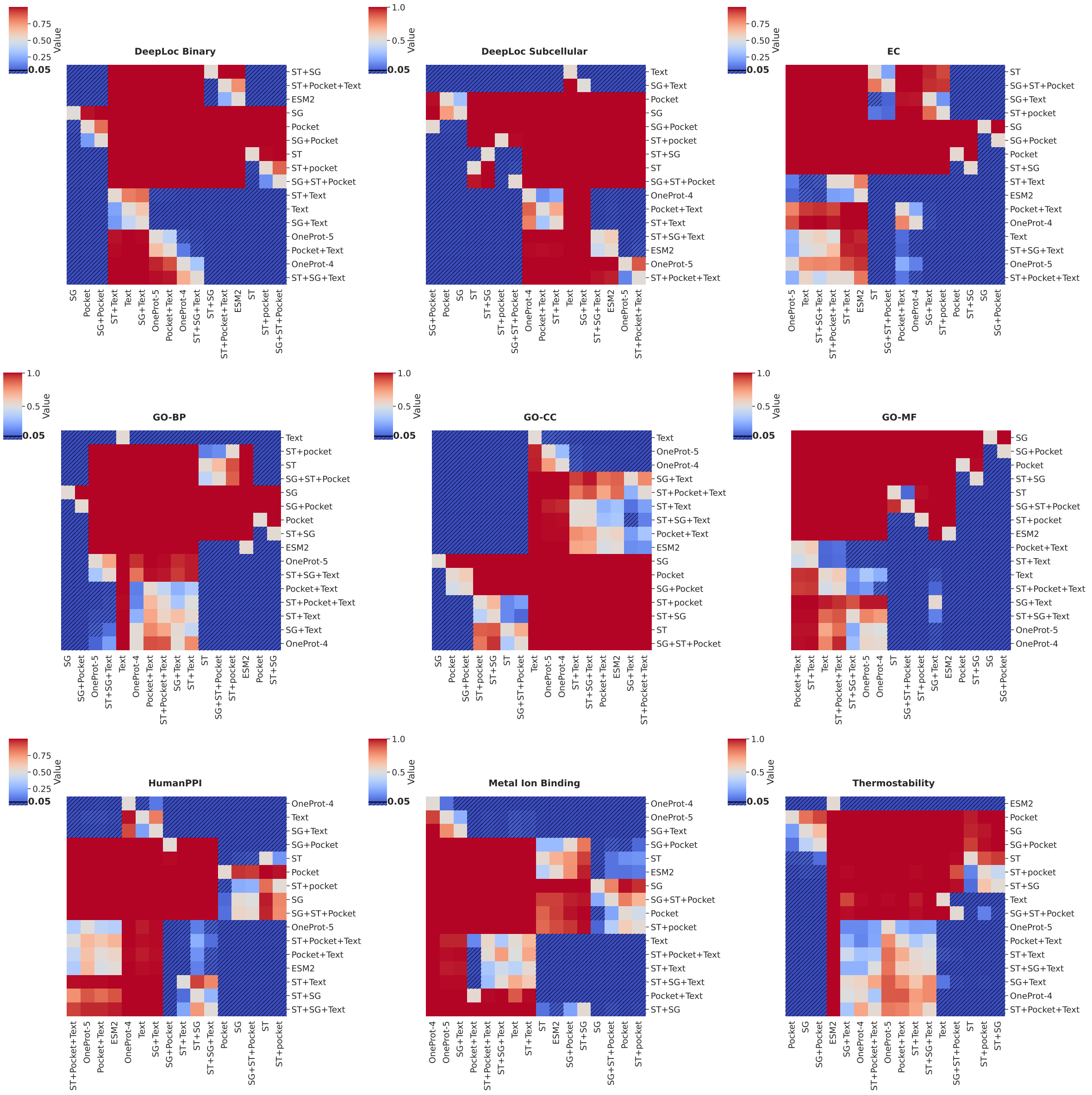

Figure S4: **Heatmaps of p-values according to one-sided Wilcoxon rank-sum test for OneProt ablations.** The alternative hypothesis of the models on the vertical axis outperforming the models on the horizontal according to metric values from Tables 3 and 4 of the main text, where for binary classification accuracy was compared. Striped pattern stands for the values $p < 0.05$, when the null hypothesis of model on the vertical axis being non-superior was rejected.
